# Genome-wide association study and KASP marker development for flour color in winter wheat

**DOI:** 10.1038/s41598-025-23358-4

**Published:** 2025-11-12

**Authors:** Yousheng Tian, Pengpeng Liu, Xin Zhang, Yichen Liu, Dezhen Kong, Yingbin Nie, Hongjun Xu, Xinnian Han, Wei Sang, Weihua Li

**Affiliations:** 1 Institute of Cotton Research, Xinjiang Academy of Agriculture and Reclamation Sciences, Shihezi, 832000 China; 2 Institute of Crop Science, Xinjiang Academy of Agriculture and Reclamation Sciences, Shihezi, 832000 China; 3 Key Laboratory of Xinjiang Production and Construction Corps for Cereal Quality Research and Genetic Improvement, Xinjiang Academy of Agriculture and Reclamation Sciences, Shihezi, 832000 China; 4 The Key Laboratory of the Oasis Ecological Agriculture, College of Agriculture, Shihezi University, Shihezi, 832003 China

**Keywords:** Wheat, Flour color, GWAS, KASP markers, Genetics, Molecular biology, Plant sciences

## Abstract

Flour color influences the quality of end-use products of common wheat (*Triticum aestivum* L.). To analyze the genetic basis of flour color, the flour brightness (FL*), red-green level (Fa*), yellow-blue level (Fb*), and whiteness (W) of 341 winter wheat materials grown during the 2019–2020 years and 2020–2021 years in Emin and Qitai were measured. A genome-wide association study was conducted using a wheat 40 K breeding chip with the MLM model. The coefficient of variation and generalized heritability of wheat flour color traits ranged from 0.62% to 22.23% and 55.52% to 83.48%, respectively. There were strong correlations across the flour color traits. GWAS identified 20 significant and stable SNP markers distributed across 16 loci, including 1 for FL* located on chromosome 5D; 6 for Fa*, located on chromosomes 1 A, 5 A, 1B (2), 6B, and 4D; 6 for Fb*, located on chromosomes 2 A (2), 4 A, 4B, 6B, and 5D; and 3 for W, located on chromosomes 2 A, 4 A, and 5D. Two KASP markers were developed for Fa*, which exhibited good genotype and significant phenotypic differences among materials with different genotypes. Seven candidate genes that may affect flour color during grain development were screened, including *TraesCS5D02G01340.1*, *TraesCS5D02G013100*, and *TraesCS5D02G014300.1* on the 5D chromosome may simultaneously influence W, FL*, and Fa*, *TraesCS1B02G269100.1* and *TraesCS1B02G269500.1* on the 1B chromosome may impact Fa*, while *TraesCS4A02G307200* and *TraesCS6B02G034100.1* on the 4 A and 6B chromosomes may affect Fb*.The results provide useful information to enhance the color quality of wheat flour in wheat.

**Supplementary Information:**

The online version contains supplementary material available at 10.1038/s41598-025-23358-4.

## Introduction

Flour color is an important characteristic in evaluating flour quality for many final product productions^1^, reflecting flour quality and milling accuracy, and serves as a significant indicator for flour grading. Therefore, it is necessary to pay attention to the study of flour color to improve the quality of wheat products and meet the market’s development needs^2^. The color of flour and its products primarily depends on the accumulation of pigment substances, such as yellow pigments and carotenoids, in the flour. Previous studies have shown that phytoene synthase (Psy)^3^, polyphenol oxidase (Ppo)^4^, lipoxygenase (Lox)^5,6^, and some other peroxidase enzymes in wheat grains affect the color, processing quality, milling quality, storage characteristics, and other quality traits of flour or flour products through the oxidative degradation of pigment substances. The yellowness of flour is mainly influenced by the quantity of carotenoids, lutein, and flavonoids^7^. The yellow pigment content in wheat flour is affected by Lox activity, which converts oxidative carotenoids to make wheat flour white^8^. Psy1 is the initial step in catalyzing carotenoid biosynthesis and serves as a crucial regulatory point, strongly correlated with carotenoid accumulation (*r* = 0.8)^9^, directly affecting the color of grain endosperm and flour. In the presence of molecular oxygen, Ppo catalyzes the oxidation of phenols to form quinones^10^, which can polymerize to create high molecular weight black or brown pigments, thereby affecting the color of wheat products, especially yellow alkaline and white salted noodles^11^. Peroxidase (POD) is a reductase that can oxidize primary phenolic acids, such as ferulic acid, leading to the formation of chromogenic groups and brown substances^12^. High POD activity may cause the flour to darken, subsequently leading to suboptimal noodle color^13^. The browning index of noodle products is significantly correlated with POD activity (*r* = 0.84–0.97)^14^.

The color of flour has a significant impact on the quality of the final wheat product. Miskelly^7^ found a significant correlation between the color of flour and the yellowing of noodles in China and Japan. Flour with a high yellow pigment content is the preferred choice for producing alkaline noodles in China and Japan. In many Asian countries, noodles are made with specially selected flour to enhance the color of the final product^15^. Therefore, yellow noodles from Japan and China require high b* value flour^16^. However, other end products, such as bread, steamed bread, dry white noodles, and dumplings, require white flour with a very low b* value. Similarly, in the United States and other parts of the world, white flour with extremely low b* values (0) and high L* values (100) is ideal for bread baking^17^.

Flour color is a quantitative trait controlled by multiple genes with high heritability^18^. Environmental and management measures may also affect the color of flour. Grain protein content, hardness, seed coat color, grain size, and shape may all contribute to changes in flour color^19^. Previous studies have reported the discovery of QTLs that affect flour color near the hardness sites (*Pina* and *Pinb*) on chromosome 5DS^2,20–22^. It is speculated that these SNP markers may also control flour color through grain hardness. At the same milling extraction rate, the flour color of low-protein wheat is whiter than that of high-protein wheat. The a* value of wheat flour with a red seed coat is higher than that of wheat with a white seed coat^19^.

Improving the color of flour is an important breeding goal for wheat. Under conventional breeding methods, the efficiency of selecting this trait is relatively low. Genetic improvement is the most effective method for enhancing the color quality of wheat. GWAS is an effective tool for understanding the genetic loci of quantitative traits and marker-assisted selection (MAS) in wheat. This study identified significant association sites for flour color through GWAS, providing a reference for future MAS breeding of flour color and the cloning of color-related genes.

## Materials and methods

### Plant materials and field trials

The association panel consists of 341 winter wheat varieties and advanced breeding lines from various wheat regions in China excluding Libellula and Strampellula (Table S1). The validation of KASP markers was conducted using 200 winter wheat materials, referred to as the validation panel in this study. This panel included various wheat varieties and advanced breeding lines from both domestic and international sources (Table S2). Among them, 193 materials were different from those in the association panel, while the other 7 materials were part of the association panel. The genotypes of the 7 materials at the KASP marker development site were known, which was used to confirm the accuracy of KASP marker genotyping. The validation panel was used to verify the success of developing KASP markers.

The association panel was evaluated at two sites, including the Institutes of Agriculture Sciences in Emin and Qitai, Xinjiang, China, during the years 2019–2020 and 2020–2021 (hereafter referred to as 2020EM, 2020QT, 2021EM, and 2021QT, respectively). The validation panel was planted in Shihezi, Xinjiang, China, during the years 2020–2021 and 2021–2022. The experiment was conducted using an alpha-lattice design with two replications. Each replication consisted of 18 incomplete blocks, with each block comprising 19 genotypes. Each genotype was grown in a plot measuring 1.8 m in length and 8 rows with 0.25 m spacing between them, each row sowed 40 seeds. Recommended management practices were applied to the trials at their respective locations. Plots were hand-harvested at maturity, and the grain was stored at 4 °C. Using the MLU202 Mill (Wuxi, China), the grain was ground and passed through a 0.1-micron sieve. These flour samples were stored in airtight containers.

### Phenotype test

The whiteness of the flour was measured using an intelligent whiteness tester (WSB-V, Zhejiang, China). Place the sample into the sample box, compact it with a pressure pad, cover it with the lid, invert the sample box, unscrew the bottom cover, remove the glass plate, and position the sample box on the measurement base for whiteness assessment.

The parameters of the colorimeter were measured using the CR-410 colorimeter (Konica Minolta, Japan), which are represented by L*, a*, and b* color spaces. L* represents brightness, where L* = 0 represents black, and L* = 100 represents white, with a total of 100 levels in between. The a* and b* values represent different color spaces. Specifically, a* represents the red-green direction, while b* represents the yellow-blue direction. In this context, +a* signifies a reddish hue, -a* indicates a greenish hue, +b* denotes a yellowish hue, and -b* signifies a bluish hue.

### Genome-Wide association study

SNP genotyping was conducted using the Wheat 40 K breeding array by the MolBreeding Company in Tianjin, China (http://www.molbreeding.com/). Markers with a minor allele frequency (MAF) below 5% and missing data exceeding 10% were excluded from further analysis. Population structure was assessed using clustering analysis software Structure v2.3.4 based on Bayesian models^23^. PCA analysis and LD analysis were performed using TASSEL 5.0^24^. The association panel was divided into two subgroups, with an LD decay distance of 4 Mb, which was detailed in our previous article^25^.

We used the MLM model in TASSEL 5.0^24^ for GWAS and utilize the population structure (Q matrix) and kinship matrix (K matrix) as covariates to prevent false positives. According to Sheoran et al.^26^, when the significance test reached *P* < 0.0001 (-log10(*P*) ≥ 4), it was determined that the marker was significantly associated with the trait. If multiple SNPs controlling a trait were identified to fall within one LD interval, they were referred to as one locus^25^. Based on previous research reports, loci associated with multiple phenotypic traits were termed pleiotropic loci, while loci consistently identified in at least two environments were considered stable loci^27^.

### Development and verification of KASP marker

Select SNPs that were significantly and stably identified in various environments for transformation into KASP markers. We utilized the online software Polymarker (http://www.polymarker.info/) to design two allele-specific forward primers and one common reverse primer. Standard FAM tags (5’ GAAGGTGAGTCATGCT 3’) and HEX tags (5’ GAAGGTCGAGTCAACGGATT 3’) were attached to the 5’ ends of the two allele-specific primers, respectively.

According to the descending thermal cycle protocol described by the manufacturer (LGC Genomics, Beverly, MA, USA), SNP genotyping was performed on an ABI7500 instrument using a 96-well plate. The genotyping outcomes were assessed using ABI7500 software, supplemented by manual assessment based on fluorescence values^28^. A *t*-test was conducted to detect significant differences in phenotypic traits between alleles. The primer pair sequences for PCR amplification are listed in Table S3.

### Identification of candidate genes

In order to identify potential candidate genes related to flour color traits, the IWGSC online database (http://www.wheatgenome.org/) was used to search for all genes within the stable SNP marker LD region, defined as 2 Mb upstream and 2 Mb downstream of the SNP flanking region, based on the Chinese spring reference genome (IWGSC RefSeq v1.0). Protein function prediction of candidate genes was performed using the UniProt protein database (https://www.uniprot.org/) and the Ensembl plants database (http://plants.ensembl.org/Triticum_aestivum/Gene). The transcriptome data from different stages of seed maturation in the publicly available Expression Atlas database (https://www.ebi.ac.uk/gxa/Experiments/E-MTAB-4484/Results) were used to study the expression characteristics of these genes^29^. Based on functional annotation, we selected genes that were highly expressed at different stages of grain maturation, as well as genes that overlap with stable SNPs for further analysis. We utilized qRT-PCR to detect the expression levels of candidate genes in 5-day, 10-day, 15-day, 20-day, 25-day, and 30-day seeds after anthesis of extreme flour color materials. The extreme color materials were “Hongzhitou” and “Yupilaina”, and their color traits are detailed in Table S4.

Total RNA was isolated from seeds at various days after anthesis using the TRIzol reagent (Invitrogen, USA), following the manufacturer’s instructions. The concentration of total RNA was measured spectrophotometrically using a NanoDrop instrument (Thermo Scientific NanoDrop 2000 C Technologies, Wilmington, USA), and the purity was assessed using the A260/A280 and A260/A230 ratios provided by NanoDrop. Reverse transcription was carried out using a PrimeScriptTM first-strand complementary DNA (cDNA) Synthesis Kit (TaKaRa, Japan).

qRT-PCR was performed using an iCycler iQTM Multicolor PCR Detection System (Bio-Rad, Hercules, CA, USA). qPCR was performed with cDNA in triplicate on 96-well plates using SYBR^®^ Premix Ex TaqTM II (TaKaRa). Each reaction (20 µL) consisted of 10 µL of SYBR^®^ Premix Ex TaqTM II, 1 µL of diluted cDNA, 0.4 µL of forward and reverse primers, and 8.2 µL of H_2_O. qPCR cycling conditions were as follows: 95 °C for 2 min, followed by 40 cycles of 95 °C for 5 s, 57 °C for 32 s. Fluorescence data were collected during the 57 °C step. Wheat Actin (Genebank ID: LOC123114174) was used as a reference gene. The gene ID and primer sequences are listed in Table S5.

### Statistical analysis

A multi-environment trial analysis was conducted using R software to perform the analysis of variance (ANOVA).

The best linear unbiased predictor (BLUP) value^30^ was calculated using the R package lme4^31^.

Broad-sense heritability (*h*^*2*^) was estimated from variance components using the formula: *h*^*2*^ = σ^2^_G_/(σ^2^_G_ + σ^2^_GE_/E + σ^2^_e_/rE), where σ^2^_G_, represents the genetic variance, σ^2^_GE_ represents the genotype × environment interaction variance, σ^2^_e_ represents the residual variance, E represents the number of environments, and r represents the number of replicates per line^32^.

Pearson’s correlation between phenotypic traits was computed using SPSS 22 (http://www.brothersoft.com/ibm-spss-statistics-469577.html).

Other ANOVA and plots were conducted in SPSS 22 and Origin 8.0, respectively.

Manhattan and Q-Q plots were created using the “qqman” package in R software^33^.

Gene expression heatmap was performed using TBtools^34^.

## Results

### Phenotyping

**Fig. 1:**
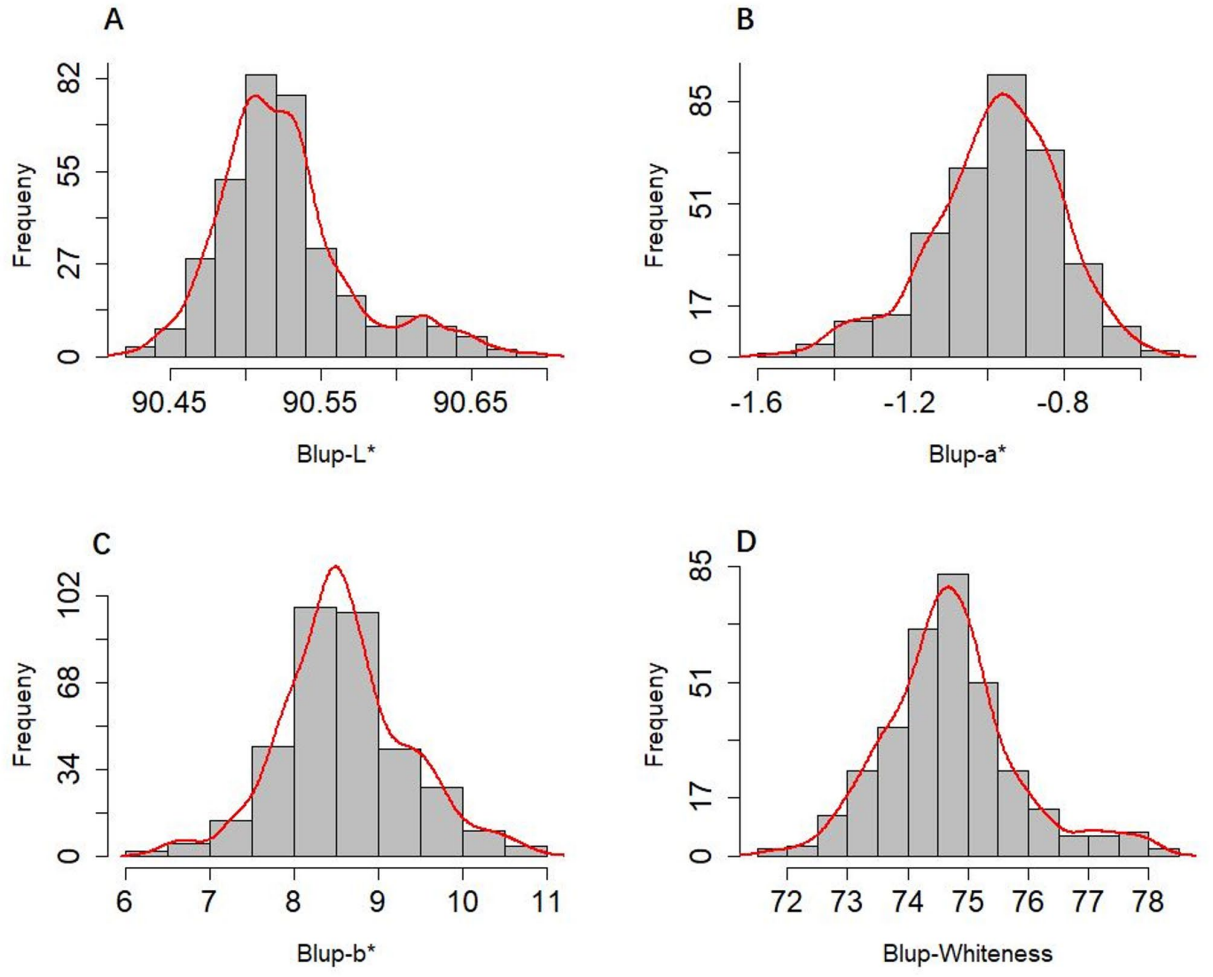
Frequency distributions of BLUP values for color traits in 341 wheat materials. (**A**), Frequency distributions of FL*; (**B**), Frequency distributions of Fa*; (**C**), Frequency distributions of Fb*; (**D**), Frequency distributions of whiteness.

Analysis of variance (ANOVA) of the association panel showed that, except for no significant difference in whiteness between environments, there were extremely significant differences in other flour color traits among genotypes, environments, and years. In addition, Fa* and Fb* showed extremely significant differences in genotype and environmental interactions, whiteness showed extremely significant differences in genotype and environmental interactions, and genotype and year interactions (Table 1). The variation ranges of FL*, Fa*, Fb*, and whiteness in different environments were 88.17–93.36, (−1.76)-(−0.34), 5.74–11.95, and 68.60–82.20, respectively. The variation ranges of coefficient variation were 0.62%−0.68%, 19.09%−22.23%, 8.36%−11.88%, and 2.25%−2.44%, respectively. The values of *h*² were 55.52%, 55.52%, 82.71%, and 83.48%, respectively (Table 2). The frequency distribution of flour color traits based on BLUP values was approximately normal (Fig. 1).

Correlation analysis was conducted on the color traits of flour based on BLUP values (Table 3). Highly significant correlations were observed between FL* and Fa*, Fb*, and whiteness, with correlation coefficients of −0.251, −0.316, and 0.738, respectively. Additionally, highly significant correlations were found between Fa* and Fb*, as well as whiteness, with correlation coefficients of −0.645 and 0.225, respectively. Furthermore, a highly significant negative correlation was identified between Fb* and whiteness, with a correlation coefficient of −0.764.

**Table 1:** Analysis of variance for color quality traits in 341 winter wheat.

Source of variance	df	Sum of squares			
		FL*	Fa*	Fb*	Whiteness
Genotypes	340	0.7^***^	0.131^***^	2.59^***^	10^***^
Environments	1	51.8^***^	8.543^***^	66.92^***^	0.5
Years	1	345.5^***^	0.254^***^	36.99^***^	2118.5^***^
Genotypes×Environments	339	0.2	0.015^**^	0.24^**^	1.2^***^
Genotypes×Years	339	0.2	0.01	0.13	1.2^***^
Residual	332	0.2	0.011	0.18	0.7

*** indicate significant differences at *P* < 0.001; ** indicate significant differences at *P* < 0.01.

**Table 2:** Phenotypic variations and heritability of color quality traits.

Trait	Environment	Minimum	Maximum	Mean	SD	CV (%)	h^2^(%)
FL^*^	2020EM	88.17	91.60	89.74	0.61	0.68	55.52
	2020QT	88.52	91.98	90.30	0.58	0.64	
	2021EM	89.19	92.70	90.94	0.56	0.62	
	2021QT	89.36	93.16	91.14	0.57	0.62	
Fa^*^	2020EM	−1.55	−0.34	−0.87	0.19	22.23	55.52
	2020QT	−1.75	−0.54	−1.06	0.22	21.19	
	2021EM	−1.45	−0.45	−0.93	0.18	19.09	
	2021QT	−1.76	−0.46	−1.05	0.23	21.42	
Fb^*^	2020EM	6.24	10.35	8.39	0.70	8.36	82.71
	2020QT	6.51	11.95	9.13	0.94	10.33	
	2021EM	6.10	10.88	8.36	0.83	9.98	
	2021QT	5.74	11.70	8.50	1.01	11.88	
Whiteness	2020EM	69.50	78.50	73.54	1.65	2.25	83.48
	2020QT	68.60	78.90	73.36	1.77	2.41	
	2021EM	71.10	81.20	75.95	1.85	2.44	
	2021QT	70.40	82.20	75.99	1.96	2.58	

2020EM, 2020QT, 2021EM, and 2021QT, the cropping seasons of 2019 ~ 2020 and 2020 ~ 2021 in E’min (EM) and Qitai (QT) respectively; SD, standard deviation; CV, coefficient of variation; *h*^2^, heritability.

**Table 3:** Phenotypic correlations (*r*) of the flour color traits for the wheat association panel.

Trait	FL^*^	Fa*	Fb*
Fa*	−0.251^**^		
Fb*	−0.316^**^	−0.645^**^	
Whiteness	0.738^**^	0.225^**^	−0.764^**^

** indicates significant differences at *P* < 0.01.

### Genome-wide association study for flour color traits

**Fig. 2:**
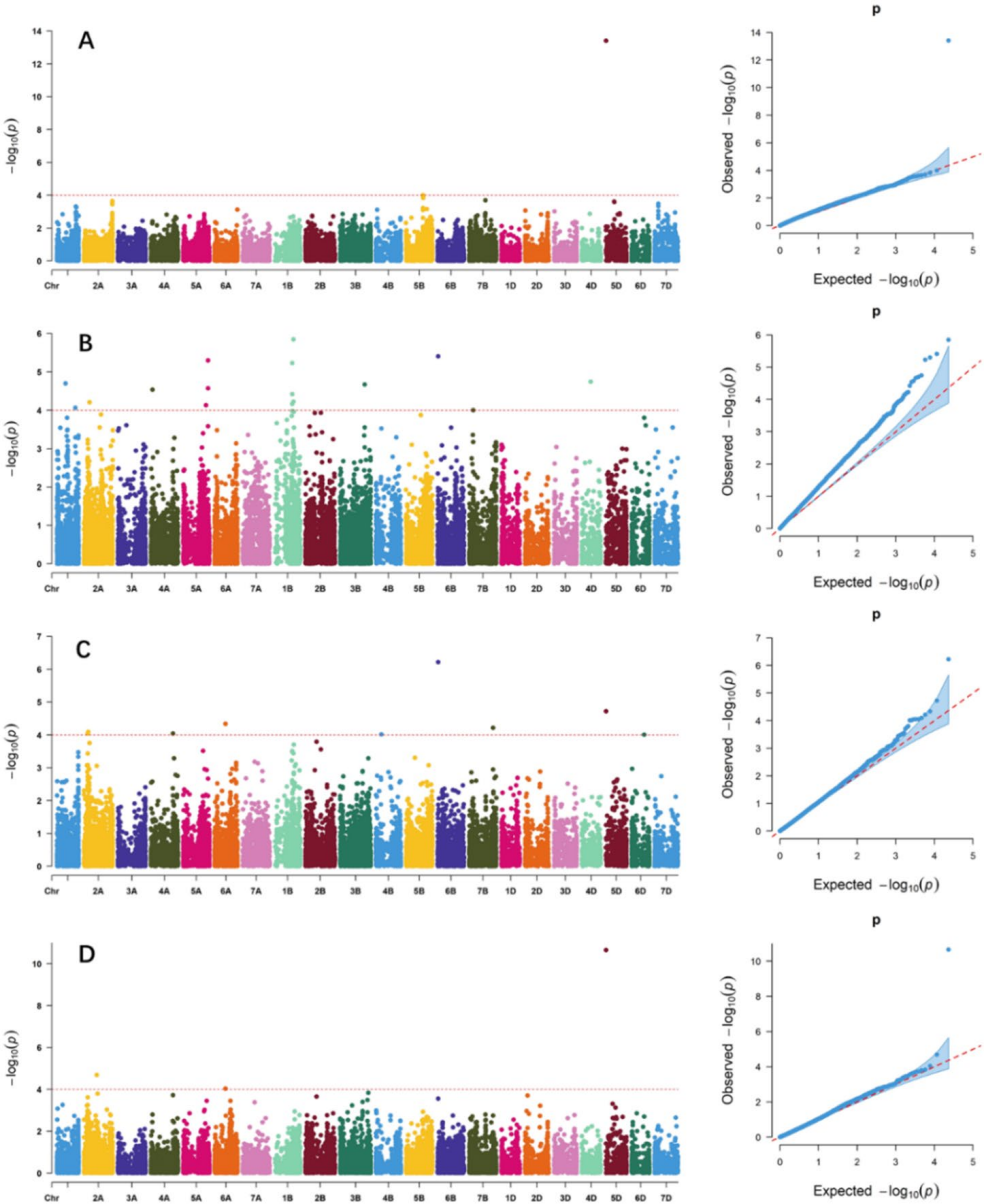
Manhattan and quantile-quantile (Q-Q) plots for color quality traits identified through genome-wide association analysis using BLUP values. (**A-D**), Manhattan, and Q-Q plots for FL*, Fa*, Fb*, and whiteness, respectively; A horizontal line represents the significance threshold at which markers were considered associated with a trait (*P* < 1E-4, = 4).

Based on BLUP values and using 23,143 SNPs after filtering out low-quality SNPs, the GWAS identified 31 marker-trait associations (MTAs) related to flour color traits (Fig. 2; Table S6), including 2 MTAs for FL*, 16 MTAs for Fa*, 10 MTAs for Fb*, and 3 MTAs for whiteness. These MTAs were distributed on chromosomes 1 A, 1B, 2 A, 3B, 4 A, 4B, 4D, 5 A, 5B, 5D, 6 A, 6B, 6D, and 7B, explaining 5.52% to 19.91% of phenotypic variation (Table S6). Simultaneously, GWAS was performed in each environment, and a total of 20 stable association signals were detected, distributed across 16 loci (Figures S1, S2, S3, S4; Table 4), including 1 locus for FL* located on chromosome 5D; 6 loci for Fa*, located on chromosomes 1 A, 5 A, 1B (2), 6B, and 4D; 6 loci for Fb*, located on chromosomes 2 A (2), 4 A, 4B, 6B, and 5D; 3 loci for whiteness, located on chromosomes 2 A, 4 A, and 5D. The marker *6B_20151350* was significantly and stably associated with both Fa* and Fb*; The marker *4A_601242583* was significantly and stably associated with both Fb* and whiteness; The marker *5D_6525346* was significantly and stably associated with FL*, Fb*, and whiteness. These markers were considered to be pleiotropic loci (Table 4).

**Table 4:** Significant single-nucleotide polymorphisms (SNPs) for flour color quality traits were identified in multiple environments.

Trait	Environment	Marker	*P*-value					*R*^2^(%)				
			E1	E2	E3	E4	E5	E1	E2	E3	E4	E5
FL*	E1 E2 E3 E4 E5	*5D_6525346*	7.12E-10	4.98E-09	2.58E-06	3.56E-05	3.87E-14	13.41	12	8.21	6.27	19.91
Fa*	E1 E2 E3 E4 E5	*1B_473486955*	2.85E-05	6.63E-05	4.67E-05	8.04E-05	5.92E-06	6.49	5.91	6.39	5.79	7.36
	E3 E5	*1B_503482454*			9.02E-05		6.01E-05			5.97		5.92
		*5A_671439068*			3.87E-05		5.06E-06			6.53		7.46
		*5A_671479200*			6.07E-05		2.67E-05			6.22		6.41
	E3 E4 E5	*6B_20151350*			4.29E-05	2.57E-05	3.93E-06			6.86	6.82	7.86
	E1 E4 E5	*1A_236720351*	1.50E-05			9.07E-06	2.01E-05	5.8			6.06	5.52
	E4 E5	*1B_474382447*				5.19E-05	3.79E-05				6.07	6.19
		*4D_238181951*				1.55E-05	1.80E-05				5.74	5.58
	E2 E5	*1B_473235903*		7.11E-05			6.87E-05		5.88			5.82
	E1 E3 E5	*1B_504760661*	1.13E-07		9.64E-06		1.43E-06	10.8		7.85		8.90
Fb*	E1 E2 E4 E5	*6B_20151350*	6.73E-06	3.94E-05		1.14E-05	6.05E-07	7.7	6.41		7.26	9.04
	E1 E3 E4 E5	*5D_6525346*	7.59E-07		1.31E-05	8.51E-05	1.90E-05	8.83		7.16	5.74	6.62
	E1 E5	*2A_104922253*	1.37E-05				8.95E-05	6.96				5.65
		*2A_117110052*	5.14E-05				8.07E-05	6.11				5.72
	E3 E5	*4A_601242583*			5.82E-05		9.00E-05			6.18		5.65
		*4B_148210072*			3.46E-05		9.67E-05			6.52		5.61
Whiteness	E1 E2 E3 E4 E5	*5D_6525346*	5.59E-12	6.41E-07	6.50E-10	1.06E-06	2.2E-11	16.66	8.83	13.85	8.56	15.57
	E1 E5	*2A_348674245*	4.43E-05				2.1E-05	6.43				7.41
	E2 E3	*4A_601242583*		8.36E-05	3.61E-05				5.73	6.47		

E1, 2020EM; E2, 2020QT; E3, 2021EM; E4, 2021QT, which represent the cropping seasons in Emin (EM) and Qitai (QT) for the years 2019 ~ 2020 and 2020 ~ 2021, respectively; E5, BLUP, the best linear unbiased predictor of protein quality traits in 341 wheat accessions during two cropping seasons across two environments.

### Development and validation of KASP markers

**Fig. 3:**
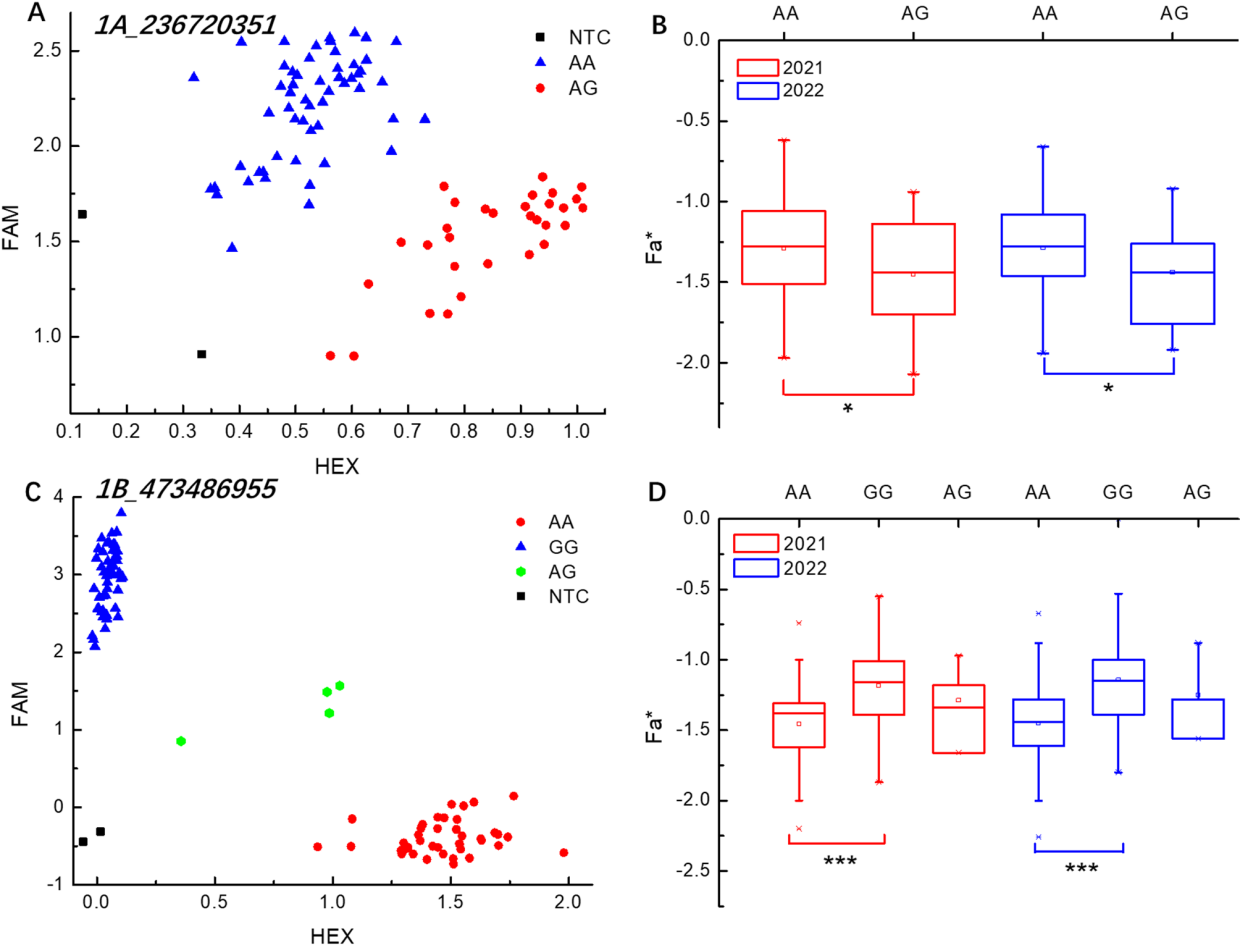
Kompetitive allele-specific PCR (KASP) verification of a significant single nucleotide polymorphism (SNP) related to the color quality. (**A**,** C**), Scatter plots of KASP markers for Fa*; (**B**,** D**), the variance of Fa* for accessions with different alleles; Red dots and blue triangles represent the homozygous genotypes, green dots represent heterozygous genotypes, and black squares on the bottom left of the plot indicate the no-template control; *** indicate significant differences at *P* < 0.001; * indicates significant differences at *P* < 0.05.

Materials were classified based on genotypes of significant and stable SNPs, and *t*-tests were used to detect the significance of genotype effects on phenotypic traits (Table S7). SNPs with highly significant genotypic effects in the four environments of 2020EM, 2020QT, 2021EM, and 2021QT were selected to develop KASP markers (Table S7). The KASP marker developed for SNP *1A_236720351* could effectively group the validation subset based on allele genotype (Fig. 3A). In this study, only AA homozygotes and AG heterozygotes were found in the validation subset, and no GG homozygotes were present. The Fa* of AA homozygous genotype was significantly higher (*P* < 0.05) than that of AG heterozygous genotype (Fig. 3B). The KASP marker developed for SNP *1B_473486955* could significantly group the materials of the AA, AG, and GG genotypes in the validation subset (Fig. 3C), and the Fa* of the GG homozygous genotype was significantly higher compared to the AA homozygous genotype (Fig. 3D).

### Candidate genes for flour color traits

SNPs that were significantly and stably associated with flour color traits, and showed significant differences in phenotype between different haplotypes across four environments (2020EM, 2020QT, 2021EM, and 2021QT; *P* < 0.001; Table S7) were selected to search for candidate genes. A total of 410 genes were detected within 2 Mb upstream and 2 Mb downstream sequences flanking those SNPs. Among them, genes before and after SNPs, and genes overlapping with SNPs were shown in Table S8, with 17 SNPs located in the intergenic region and 3 SNPs located within genes. GO enrichment analysis showed that these genes were involved in a total of 439 GO terms, classified into 15 biological processes, 2 cellular components, and 10 molecular functions. Most genes were mainly concentrated in metabolic processes and cellular processes of biological processes, cellular anatomical entities and protein-containing complexes of cellular components, and binding and catalytic activities of molecular functions (Figure S5C). KEGG^35–37^ analysis showed that these genes were mainly enriched in metabolic pathways, biosynthesis of secondary metabolites, steroid biosynthesis, photosynthesis, oxidative phosphorylation, ribosome, and other processes (Figure S5B).

**Fig. 4:**
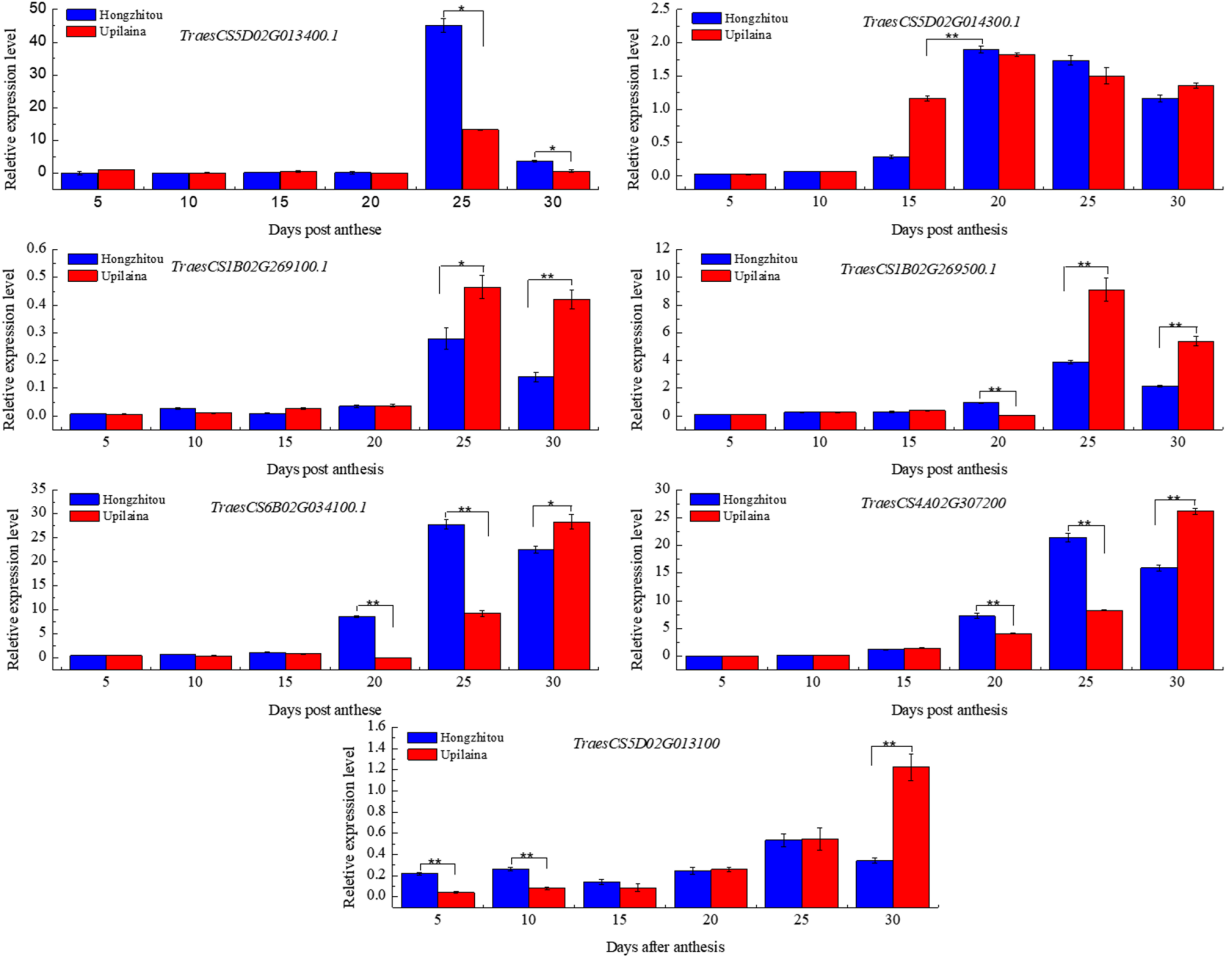
Expression of candidate genes in seeds of extreme flour color quality materials at 5, 10, 15, 20, 25, and 30 days after flowering. HZT indicates “Hongzhitou”; YPLN indicates “Youpilaina”.

Based on the RNA-seq data from a public expression database and functional annotations of those genes, and also considering genes that overlap with significant SNPs, the present study selected 7 candidate genes for qRT-PCR analysis. The relative quantitative data were transformed by log10, and a heatmap was drawn (Figure S5A). Six candidate genes were differentially expressed in seeds of flour color extreme materials (Fig. 4; Table S9). *TraesCS5D02G013100* encodes PMA1, which was differentially expressed in 30-day seeds after anthesis of extreme materials; *TraesCS5D02G014300.1* encodes P450, which was differentially expressed in 15-day seeds after anthesis of extreme materials; *TraesCS1B02G269500.1* encodes isopentenyl diphosphate delta isomerase, which was differentially expressed in 25-day and 30-day seeds after anthesis of extreme materials; *TraesCS6B02G034100.1* encodes the DExH-box ATP-dependent RNA helicase DExH12, which was differentially expressed in 20-day, 25-day, and 30-day seeds after flowering of extreme materials; This study did not annotate the protein function of gene *TraesCS4A02G307200*, which was differentially expressed in seeds of extreme materials at 20, 25, and 30 days after flowering; *TraesCS1B02G269100.1* encodes glutathione transferase, which was differentially expressed in 25-day and 30-day seeds of materials after flowering (Fig. 4; Table S9).

## Discussion

### Analysis of flour color traits

The whiteness and color-related traits of flour are crucial factors that determine the quality of the final wheat product. Therefore, it is essential to identify the main and stable allele loci for these traits and then transfer these favorable alleles to the commodity variety^2^. In this study, significant differences were observed in the flour color traits among different genotypes, environments, and years, except for whiteness among different environments (Table 1), indicating that flour whiteness was mainly controlled by genetic makeup rather than environmental factors^38^. Flour whiteness and FL* showed high heritability in this study (Table 2), suggesting that these traits were primarily controlled by genetics, making them easier to improve and breed at the genetic level^39^. All color traits exhibited a continuous distribution in the association panel (Fig. 1), displaying typical quantitative trait characteristics, indicating that they were controlled by polygenes, consistent with previous report^2^. Correlation analysis revealed highly significant correlations between flour color traits (Table 3), which align with previous research results^2,22,39,40^.

### Genome-wide association study for FL*

This study consistently identified one locus on chromosome 5D (*5D_6525346*) significantly associated with FL*, explaining the highest phenotypic variation rate of 19.91%. In line with the finding of Chen et al.^41^, who pinpointed a significant marker *wPt-0853-cfd18* associated with FL* at 5,597,656 bp on chromosome 5D. Previous research has also identified the primary QTL/genes influencing FL* on chromosomes 2 A, 3 A, 4 A, 6 A, 7 A, 1B, 3B, 4B, 5B, 7B, 2D, 4D, and 5D^2,42–46^. For instance, Schmidt et al.^45^ identified a QTL associated with FL* on chromosome 4B (*R*^2^ = 5%). Johnson et al.^44^ reported that QTLs for FL* were located on chromosome 2 A (189.8 cM) and 6 A (0.1–3.1.1.1 cM). Zhao et al.^46^ detected 13 QTLs for FL* on chromosomes 1 A (5 cM), 6 A (1–5 cM), 1B (3–4 cM), 2B (10 cM), 3B (2 cM), 5B (9–11 cM), 7B (cM), 2D (2, 8, 15 cM), and 5D (0 cM) using the recombinant inbred line ‘Chuan 35050 × Shannong 483’ as the material.

### Genome-wide association study for Fa*

This study identified 6 loci significantly and stably associated with Fa* on chromosomes 1 A, 5 A, 1B, 6B, and 4D (Table 4). Similarly, previous studies identified QTL/genes linked to flour Fa* on chromosomes 1 A, 3 A, 4 A, 6 A, 7 A, 3B, 5B, 6B, 7B, 4D, 5D, and 7D^2,42,44^. Zhao et al.^46^ identified 12 QTL/genes associated with Fa* on chromosomes 3 A (20 cM), 5 A (30 cM), 6 A (0 and 6 cM), 1B (0 cM), 2B (1, 4, 11 cM), 4B (11 cM), 6B (4 cM), 7B (12 cM), and 5D (0 and 2 cM). Zhang et al.^39^ used 240 recombinant inbred lines (RILs) derived from crossing the Chinese wheat variety PH82-2 with Neixiang 188 to map the QTLs of Fa* on chromosome 1 A (*Xwmc120 - Xbarc269*), 4 A (*Xwmc468 - Xbarc170*), 7 A (*Xwmc809 - YP7A*), 1B (*Sect. 1 - HVM23*), and 3B (*Xbarc84 - Xbarc77*), explaining up to 35.9% of the phenotypic variation. Additionally, Zhang et al.^40^ employed 168 diploid (DH) lines hybridized with Huapei 39 and Yumai 57 to identify a major QTL *qa1B* for Fa* on chromosome 1B (0.1 cM), which accounts for 25.64% of the phenotypic variation.

### Genome-wide association study for Fb*

The yellowness index (Fb*) of flour was mainly influenced by the quantity of carotenoids, luteins, and flavonoids^7^. This study identified 6 loci significantly associated with Fb* on chromosomes 2 A, 4 A, 4B, 6B, and 5D (Table 4). Johnson et al.^44^ conducted GWAS on 243 varieties and advanced breeding lines selected from the past 20 years and identified loci significantly associated with yellowing on chromosomes 4 A (159.5 cM), 4B (22.5–26.4 cM), and 7B (120.4–123.2 cM and 138.3–140.4 cM). Mares and Campbell^47^ identified QTLs associated with Fb* mapped on chromosome 7 A in two populations of three diploid Australian wheat populations. Schmidt et al.^45^ identified two QTLs associated with Fb* on chromosomes 3 A (*R*^2^ = 5%) and 4B (*R*^2^ = 12%). Kuchel et al.^48^ identified the main QTL for Fb* on chromosome 7B (*Xgwm283 - Xgwm146*), explaining up to 77% of phenotypic variation. Zhao et al.^46^ detected 13 QTLs for Fb* on chromosomes 1 A (0, 2, 3, and 7 cM), 4 A (4 cM), 6 A (0, 1, and 4 cM), 1B (0 cM), 5B (1 and 3 cM), 7B (0 cM), 2D (12 cM), 4D (9 cM), 5D (9 cM), and 6D (0 cM). Parker et al.^49^ reported two QTLs on chromosomes 3 A (*Xbcd828*−3 A) and 7 A (*Xcdo347-7 A*, *Xcdo347-7 A*), explaining 60% and 13% of Fb* total phenotypic variation, respectively. Zhang et al.^42^ detected the main QTL of Fb* on chromosome 7 A, accounting for 12.1% to 37.6% of phenotypic variation in five environments. Zhang et al.^39^ used 240 recombinant inbred lines (RILs) obtained by crossing the Chinese wheat variety PH82-2 with Neixiang 188 to map the QTL of Fb* on chromosomes 7 A (*Xwmc809-YP7A*) and 1B (*Sect. 1-HVM23*). In summary, loci associated with Fb were detected multiple times on chromosomes 1B, 3 A, 7 A, and 7B; however, they were not identified in our study. This discrepancy may be due to the different SNP chip and associated panel utilized in this research compared to previous studies. The loci identified in this study on chromosomes 2 A and 6B have not been reported and may be new genetic loci related to Fb*.

### Genome-wide association study for flour whiteness

This study identified three loci significantly and stably associated with flour whiteness on chromosomes 2 A, 4 A, and 5D (Table 4). Among these, the locus on chromosome 5D (*5D_6525346*) was consistently detected in all environments, with the highest phenotype interpretation rate of 16.66%. In line with previous research, Ji et al.^2^ found a significant SNP *BS0000020_51* associated with flour whiteness in multiple environments, with the highest phenotypic variation explanatory rate of 15.95%. Notably, this locus was only 3 Mb away from the SNP (*5D_6525346*) identified in this study.

Notably, SNP marker *5D_6525346* was detected to be significantly associated with flour FL*, Fb*, and whiteness (Table 4). Previous studies have also detected QTLs associated with flour color near this marker, indicating that there might be important genes affecting flour color at this locus^50^. The whiteness of flour was also affected by the grinding characteristics^51^. However, the grinding characteristics are influenced by particle hardness^52,53^, which in turn affects the whiteness and color of flour^2^. *Pin A* and *Pin B* were genes related to grain hardness, and any deletion or mutation in a gene can lead to hardness; *Pinb-D1* has multiple types of mutations4^7,54^. Tsilo et al.^21^ found a QTL associated with Fb* and FL* on the chromosome 5D, which was consistent with the hardness (Ha) site reported by Matter et al.^20^ on the 5DS. Zhai et al.^22^ identified a distance of 2.1 cM between QTL *QFL.caas-5D-1* and the *Pin-b* gene. The SNP marker *BS0000020_51* identified by Ji et al.^2^ on the 5D chromosome was significantly associated with flour whiteness, FL*, and Fb*. Compared with the results of Zhai et al.^22^, it was inferred that this SNP marker may also control flour color through grain hardness. This study screened the marker *5D_6525346* for color association analysis, with a distance of 3.5 Mb from the *Pinb-D1* (chromosome 5D, 3,031,551-3,032,419) gene, indicating that *Pinb-D1* might also affect flour color, which was consistent with the research results of Zhai et al.^55^.

### Development of KASP markers

SNPs are currently the most widely used molecular markers because they are ubiquitous in a given genome and have lower costs compared to other marker technologies^56^. KASP uses endpoint fluorescence detection to distinguish labeled alleles, making it an advanced SNP genotyping technique^57^. If multiple SNPs are identified within the same LD interval, theoretically only one SNP needs to be developed as a KASP marker. However, considering that some SNPs may be located in regions where designing good primers is not possible, or the genotyping of some primers may not be optimal, or the phenotype difference is not significant even though the genotyping might be clear. Therefore, multiple SNPs can be selected for KASP transformation simultaneously, and ultimately, the KASP marker with good genotyping and a significant phenotype difference can be chosen for breeding. This study developed two KASP markers from significant and stable SNPs associated with Fa* identified by GWAS (Fig. 3). Among them, the KASP marker transformed from *1B_473486955* was genotyping distinct, and notable phenotypic differences were observed among different haplotypes, which can be utilized for marker-assisted selection breeding.

### Identification of candidate genes

Previous studies have reported that the color of flour mainly depends on the accumulation of pigment substances such as yellow pigments and carotenoids in flour, as well as the oxidative degradation of pigment substances by some other peroxidase enzymes (polyphenol oxidase, peroxidase, lipoxygenase, etc.)^18^. The yellow pigment content in wheat flour is influenced by the activity of lipoxygenase (LOX), which catalyzes the oxidation of carotenoids, resulting in white wheat flour^58^. The primary gene responsible for LOX activity in wheat was located at the *TaLox-B1* locus on chromosome 4BS^59^. Complementary dominant functional markers, *LOX16* and *LOX18*, have been developed by Geng et al.^60^ based on this locus and have been extensively used to detect LOX activity in wheat germplasm resources^61^. This research identified the candidate gene Lipoxygenase LOX2.1, associated with whiteness, on the 5D chromosome through GWAS (Fig. 4; Table S7). This gene may represent a novel gene that influences wheat LOX activity, warranting further investigation.In addition, *TraesCS5D02G013100* encodes the plasma membrane H+-ATPase (LHA1), which is the primary pump responsible for establishing the plant cell membrane potential. This enzyme not only governs fundamental plant cell functions but also plays a role in responding to diverse environmental stimuli and signaling events^62^. *TraesCS5D02G014300.1* encodes a plant cytochrome P450, which plays a crucial role in many biosynthetic pathways, particularly those involving the production of multiple secondary metabolites^63^. *TraesCS1B02G269100.1* encodes glutathione transferase, which is an ancient, multi-member, and diverse enzyme class. Plant glutathione transferase has multiple effects on plant development, endogenous metabolism, stress tolerance, and exogenous detoxification^64^. *TraesCS1B02G269500.1* encodes isopentenyl diphosphate delta isomerase. Plant hormones (such as abscisic acid, gibberellin, and cytokinin), plant alcohols, carotenoids, and monoterpenes are obtained through the plastid non-methylhydroxyvaleric acid pathway^65^. Isopentenyl diphosphate serves as a common precursor for all isoprenoids in this pathway^66^. *TraesCS6B02G034100.1* encodes the DExH box ATP-dependent RNA helicase DExH12 (BRR2a). In Arabidopsis, a missense mutation in BRR2a leads to splicing defects in FLC (Flowering Locus C), resulting in reduced FLC transcript levels and premature flowering^67^. The function of *TraesCS4A02G307200* remains unknown based on the available database information. Further investigation is necessary to understand the roles of these genes in flour coloration.

## Conclusion

The coefficient of variation of flour color traits ranged from 0.62% to 22.23%, and the generalized heritability ranged from 0.56 to 0.83. They all follow an approximately normal distribution. There were highly significant correlations between flour color traits. GWAS identified 20 SNP markers significantly and stably associated with flour color traits, spread across 16 loci, including 1 for FL*, 6 for Fa*, 6 for Fb*, and 3 for whiteness. Two KASP markers were successfully developed for Fa*. Seven candidate genes that may influence flour color were identified. This study provides valuable information on genes or genetic loci related to flour color and also offers KASP markers for marker-assisted selection to enhance wheat flour color quality in China.

## Supplementary Information

Below is the link to the electronic supplementary material.

Supplementary Material 1

## Data Availability

The data presented in the study are deposited in Figshare DOI: https://doi.org/10.6084/m9.figshare.26360368.
