# Supplementary material for: Genome-wide association study and KASP marker development for flour color in winter wheat
Source: Sci Rep. 2025 Nov 12;15:39673. doi: 10.1038/s41598-025-23358-4 (PMC12612271; doi:10.1038/s41598-025-23358-4)
Supplement: Supplementary file 1 — Supplementary Material 1 [file 41598_2025_23358_MOESM1_ESM.docx]

Table S1 The names of 341 accessions in the association panel

| Code | Name | Code | Name | Code | Name |
| --- | --- | --- | --- | --- | --- |
| 1 | Xindong 18 | 115 | 2013(86)/2-3-1 | 229 | YY-103/2 |
| 2 | Xindong 2 | 116 | 2013(86)/7-1-1 | 230 | YY-120/2 |
| 3 | Xindong 14 | 117 | 2013(94)/3-2-1 | 231 | YY-121/2 |
| 4 | Xindong 15 | 118 | 2013(94)/3-2-2 | 232 | YY-122/3 |
| 5 | Xindong 16 | 119 | 2013(98)/4-4-2 | 233 | YY-123/2 |
| 6 | Xindong 17 | 120 | 2013(104)/1-1-2 | 234 | YY-125/1 |
| 7 | Xindong 18 | 121 | 2013(104)/1-2-1 | 235 | YY-126/1 |
| 8 | Xindong 19 | 122 | 2013(108)/1-1-1 | 236 | YY-138/1 |
| 9 | Xindong 20 | 123 | 2013(110)/2-6-2 | 237 | YY-139/3 |
| 10 | Xindong 21 | 124 | 2013(127)/2-1-1 | 238 | YY-142/1 |
| 11 | Xindong 22 | 125 | 2013(130)/2-1-3 | 239 | YY-234/2 |
| 12 | Xindong 23 | 126 | 2013(130)/2-3-2 | 240 | M-3/4 |
| 13 | Xindong 24 | 127 | 2013(130)/2-4-1 | 241 | Shidong 03112 |
| 14 | Xindong 26 | 128 | 2013(133)/3-2-2 | 242 | Shidong 0358 |
| 15 | Xindong 27 | 129 | 2013(143)/1-3-2 | 243 | Shidong 0821 |
| 16 | Xindong 28 | 130 | 2013(148)/2-3-1 | 244 | Shidong 04104 |
| 17 | Xindong 29 | 131 | 2013(148)/5-2-2 | 245 | Shidong 01162 |
| 18 | Xindong 30 | 132 | 2013(154)/1-1-2 | 246 | Shidong 0349 |
| 19 | Xindong 31 | 133 | 2013(154)/4-1-1 | 247 | Shidong 0451 |
| 20 | Xindong 32 | 134 | 2013(158)/1-2-1 | 248 | Shidong 03194 |
| 21 | Xindong 33 | 135 | 2013(178)/1-4-1 | 249 | Shidong 06510 |
| 22 | Xindong 36 | 136 | 2013(178)/8-3-1 | 250 | JSH15-08 |
| 23 | Xindong 37 | 137 | 2013(183)/3-1-1 | 251 | JSH606 |
| 24 | Xindong 38 | 138 | 2013(190)/6-2-1 | 252 | JSH15-06 |
| 25 | Xindong 40 | 139 | 2013(202)/3-1-1 | 253 | Xingmu1602 |
| 26 | Xindong 41 | 140 | 2013(205)/5-1-1 | 254 | Xingmu 1603 |
| 27 | Xindong 42 | 141 | 2013(206)/2-1-1 | 255 | Fengyuandong 3 |
| 28 | Xindong 44 | 142 | 2013(223)/3-2-2 | 256 | 0858 |
| 29 | Xindong 45 | 143 | 2013(230)/6-4-2 | 257 | SDWW-7 |
| 30 | Xindong 48 | 144 | 2013(230)/8-2-1 | 258 | 03--40 |
| 31 | Xindong 49 | 145 | 2013(230)/10-1-3 | 259 | 08/16 |
| 32 | Xindong 51 | 146 | 2013(230)/11-2-1 | 260 | 04--1 |
| 33 | Xindong 52 | 147 | 2013(247)/5-1-1 | 261 | Xinniang 14-6151 |
| 34 | Xindong 53 | 148 | 2013(247)/5-4-2 | 262 | Xinniang 15-2217 |
| 35 | Xindong 54 | 149 | 2013(251)/1-1-1 | 263 | Xinniang 606 |
| 36 | Xindong 55 | 150 | 2013(251)/5-3-1 | 264 | 2015J/176 |
| 37 | Xindong 57 | 151 | 2013(255)/2-4-1 | 265 | HWX1109 |
| 38 | Xindong 60 | 152 | 2013(255)/6-1-1 | 266 | 09BC-3 |
| 39 | Shidong 7 | 153 | 2013(255)/8-4-2 | 267 | Zhonglumai 666 |
| 40 | Shidong 8 | 154 | 2013(255)/10-1-2 | 268 | JFM-2 |
| 41 | Shidong 9 | 155 | 2013(255)/11-2-1 | 269 | Xm1607 |
| 42 | Yinong 16 | 156 | 2013(255)/11-2-3 | 270 | CA15026 |
| 43 | Yinong 17 | 157 | 2013(255)/11-2-5 | 271 | BH5335 |
| 44 | Kuidong 4 | 158 | 2013(255)/11-2-7 | 272 | 0868 |
| 45 | Kuihua 1 | 159 | 2013(255)/11-3-2 | 273 | 0818 |
| 46 | 95（20）/8-1-2 | 160 | 2013(255)/11-8-2 | 274 | Xinniang 152148 |
| 47 | 99（55）/3-1-1 | 161 | 2013(255)/11-9-2 | 275 | Xinniang 152183 |
| 48 | 99（79）/2-1-2 | 162 | 2013(255)/12-2-8 | 276 | JHD06 |
| 49 | 2000(125)/4-2-1 | 163 | 2013(255)/12-5-1 | 277 | JHD09 |
| 50 | 2000（125）/4-2-2 | 164 | 2013(256)/1-2-3 | 278 | 2012J176 |
| 51 | 99AR142-1 | 165 | 2013(256)/4-1-1 | 279 | LFM1512 |
| 52 | 99AR144-1 | 166 | 2014(6)/9-1-2 | 280 | LFM1430 |
| 53 | 2009(6)/8-1-3 | 167 | 2014(13)/2-2-1 | 281 | 99-5091 |
| 54 | 2009(67)/3-3-1 | 168 | 2014(13)/5-1-2 | 282 | CA12107 |
| 55 | 2009(126)/10-2-1 | 169 | 2014(13)/5-1-5 | 283 | GZ1815 |
| 56 | 2009(172)/2-2-1 | 170 | 2014(14)/1-1-1 | 284 | GZ1820 |
| 57 | 2010(27)/6-5-1 | 171 | 2014(14)/6-1-2 | 285 | CA13012 |
| 58 | 2010(48)/1-2-1 | 172 | 2014(17)/10-1-2 | 286 | CA16125 |
| 59 | 2010(60)/4-4-3 | 173 | 2014(20)/8-1-2 | 287 | CA17097-1 |
| 60 | 2010(101)/4-2-3 | 174 | 2014(20)/11-2-1 | 288 | 16GZ1612 |
| 61 | 2010(112)/6-2-2 | 175 | 2014(35)/6-1-2 | 289 | Tangshan 6898 |
| 62 | 2010(126)/3-1-2 | 176 | 2014(37)/5-3-1 | 290 | Jimai 24 |
| 63 | 2010(162)/8-2-2 | 177 | 2014(71)/3-3-1 | 291 | Jimai 26 |
| 64 | 2010(229)/1-2-2 | 178 | 2014(73)/4-1-1 | 292 | Jimai 31 |
| 65 | 2011(65)/1-1-1 | 179 | 2014(73)/4-1-2 | 293 | Han 5316 |
| 66 | 2011(70)/4-1-4 | 180 | 2014(75)/8-2-1 | 294 | Han 6172 |
| 67 | 2011(275)/2-1-1 | 181 | 2014(75)/8-2-2 | 295 | Heniang 12 |
| 68 | 2012(27)/7-2-4 | 182 | 2014(75)/15-1-2 | 296 | Shixin 618 |
| 69 | 2012(27)/7-6-1 | 183 | 2014(85)/2-2-1 | 297 | Liangxing 66 |
| 70 | 2012(41)/1-2-2 | 184 | 2014(86)/5-2-3 | 298 | Liangxing 99 |
| 71 | 2012(80)/10-1-1 | 185 | 2014(94)/2-2-2 | 299 | Jimai 17 |
| 72 | 2012(82)/7-4-3 | 186 | 2014(95)/1-2-1 | 300 | Jimai 19 |
| 73 | 2012(84)/3-3-4 | 187 | 2014(123)/1-1-1 | 301 | Jimai 20 |
| 74 | 2012(84)/3-5-2 | 188 | 2014(123)/1-2-1 | 302 | Jimai 21 |
| 75 | 2012(103)/3-2-2 | 189 | 2014(131)/1-2-1 | 303 | Jimai 22 |
| 76 | 2012(104)/3-1-2 | 190 | 2014(135)/2-1-3 | 304 | Jimai 23 |
| 77 | 2012(107)/8-1-4 | 191 | 2014(151)/5-2-3 | 305 | Jimai 44 |
| 78 | 2012(111)/5-2-6 | 192 | 2014(156)/2-1-3 | 306 | Jimai 229 |
| 79 | 2012(162)/4-1-3 | 193 | 2014(174)/2-1-1 | 307 | Yannong 19 |
| 80 | 2012(214)/2-1-1 | 194 | 2014(180)/2-2-3 | 308 | Taishan 241 |
| 81 | 2012(17)/3-1-4 | 195 | 2014(184)/2-2-2 | 309 | Yannong 173 |
| 82 | 2012(41)/1-2-2 | 196 | 2014(184)/2-3-2 | 310 | Shannong 22 |
| 83 | 2012(68)/8-1-1 | 197 | 2014(186)/2-2-1 | 311 | Yan 1212 |
| 84 | 2012(80)/10-1-3 | 198 | 2014(191)/3-2-1 | 312 | Yumai 34 |
| 85 | 2012(82)/7-5-1 | 199 | 2014(191)/3-2-3 | 313 | Gaocheng 8901 |
| 86 | 2012(202)/4-1-2 | 200 | 2014(194)/14-2-1 | 314 | Zheng 366 |
| 87 | 2013(27)/8-5-1 | 201 | 2014(197)/1-2-2 | 315 | Zhengmai 379 |
| 88 | 2013(206)/2-1-1 | 202 | 2014(199)/1-2-1 | 316 | Zhengmai 7698 |
| 89 | 2011(70)/4-1-4 | 203 | 2014(200)/1-1-3 | 317 | Zhengmai 9023 |
| 90 | 2012(67)/9-1-2 | 204 | 2014(205)/10-1-2 | 318 | Zhoumai 18 |
| 91 | 2013(37)/5-4-1 | 205 | 2014(205)/10-2-2 | 319 | Zhoumai 19 |
| 92 | 2013(62)/2-5-1 | 206 | 2014(214)/2-1-2 | 320 | Zhoumai 21 |
| 93 | 2013(69)/4-1-2 | 207 | 2014(229)/7-1-1 | 321 | Zhoumai 22 |
| 94 | 2013(85)/2-1-1 | 208 | 2014(230)/7-2-2 | 322 | Zhoumai 27 |
| 95 | 2013(115)/1-5-1 | 209 | 2014(233)/2-1-1 | 323 | Zhoumai 28 |
| 96 | 2013(123)/1-1-1 | 210 | 2014(235)/5-1-1 | 324 | Zhoumai 30 |
| 97 | 2013(148)/4-1-2 | 211 | 2013(28)/8-1-1 | 325 | Zhoumai 32 |
| 98 | 2013(154)/2-3-1 | 212 | 2013(39)/4-1-1 | 326 | Fengdecunmai 5 |
| 99 | 2013(154)/4-1-3 | 213 | 2013(39)/5-2-2 | 327 | Guan 35 |
| 100 | 2013(178)/1-3-1 | 214 | 2013(39)/5-5-3 | 328 | Aikang 58 |
| 101 | 2013(223)/5-3-1 | 215 | 2013(39)/11-4-1 | 329 | Xiaoyan 22 |
| 102 | 2013(227)/1-1-1 | 216 | 2013(39)/15-1-1 | 330 | Xiaoyan 54 |
| 103 | 2013(227)/1-1-2 | 217 | 2013(39)/15-3-2 | 331 | Kemai 1 |
| 104 | 2013(230)/11-3-1 | 218 | FZ-35/2 | 332 | Xinong 979 |
| 105 | 2013(243)/5-2-1 | 219 | FZ-46/5 | 333 | Shinuan 02-1 |
| 106 | 2013(247)/5-2-1 | 220 | FZ-48/2 | 334 | Zhongyou 206 |
| 107 | 2013(248)/5-1-1 | 221 | FZ-50/4 | 335 | Zhongyou 9507 |
| 108 | 2013(13)/4-1-1 | 222 | FZ-59/1 | 336 | Y119 |
| 109 | 2013(23)/4-1-1 | 223 | YY-2/1 | 337 | Jing 411 |
| 110 | 2013(27)/8-4-1 | 224 | YY-22/4 | 338 | 03-6118 |
| 111 | 2013(27)/8-5-1 | 225 | YY-57/3 | 339 | compton/yangmai 158 |
| 112 | 2013(58)/7-2-1 | 226 | YY-58/1 | 340 | Libellula |
| 113 | 2013(69)/4-1-1 | 227 | YY-59/1 | 341 | Strampellula |
| 114 | 2013(79)/3-1-1 | 228 | YY-84/2 |  |  |

All of the accessions originate from different provinces in China.

Table S2 The names of 200 accessions in the validation panel

| Code | Name | Code | Name |
| --- | --- | --- | --- |
| DZ001 | Hongzhitou 10 | DZ312 | Heisuidongmai |
| DZ028 | Yinong 18 | DZ313 | Fengchan 3 |
| DZ029 | Yinong 19 | DZ314 | Shijiazhuang 54 |
| DZ030 | Yinong 20 | DZ317 | Jinmai 22 |
| DZ031 | Yinong 21 | DZ320 | Jingzuo 210 |
| DZ032 | Kuidong 4 | DZ322 | F42-7 |
| DZ034 | Kuihua 2 | DZ326 | Duli |
| DZ039 | 80-453 | DZ327 | Xiusidun |
| DZ040 | 85(1) | DZ329 | Botre |
| DZ041 | 86(46)/0-2-5-3-1 | DZ333 | Quzishi21323 |
| DZ043 | 87YF5 | DZ337 | 93-410 |
| DZ044 | 89(100) | DZ348 | 92-5 |
| DZ045 | 89-(20)-1-2 | DZ356 | Lanketa |
| DZ046 | 89(34) | DZ358 | Athas66 |
| DZ047 | 89(35) | DZ370 | Lanlixiaomai |
| DZ048 | 89-117 | DZ384 | Xiaoyan 54 |
| DZ049 | 89-20/2 | DZ386 | Zhongyin 85 |
| DZ050 | 89-44 | DZ411 | Shi 02-6207 |
| DZ051 | 89-813 | DZ419 | Bainong 68 |
| DZ052 | 91(28) | DZ444 | Lankao 906-4 |
| DZ053 | 91-8 | DZ445 | 5R624（Lr4/C9355） |
| DZ054 | 96254-1 | DZ447 | 5R623 |
| DZ055 | 99-5019 | DZ448 | 5R619（Lr38） |
| DZ056 | 0118 | DZ453 | Zhou 8425B |
| DZ058 | 05/2111 | DZ462 | Shi 02-5289 |
| DZ060 | Xindong 39 | DZ465 | 035303 |
| DZ061 | 06/6155 | DZ467 | 0138 |
| DZ062 | D04-L4 | DZ468 | Huaimai 0208 |
| DZ064 | Jintianshan 8 | DZ469 | Nongda 3291 |
| DZ068 | 98(110)/5-3-1 | DZ473 | Keyi 4058 |
| DZ069 | 98(101)/13-3 | DZ478 | Shannong 2149 |
| DZ071 | 96(60)/3-1-3 | DZ480 | CA0391-1 |
| DZ076 | 98(111)/5-2-2 | DZ487 | Shan 253 |
| DZ086 | 98(21)/2-2-3 | DZ494 | Jingdong 8 |
| DZ087 | 99(55)/5-2 | DZ500 | Shijiazhuang 8 |
| DZ089 | 98(78)/9-3-1 | DZ501 | Shimai 15 |
| DZ093 | 98(83)/2-1-1 | DZ506 | Shi 4185 |
| DZ095 | 98(107)/1-3-1 | DZ507 | Heng 5229 |
| DZ098 | 98(107)/3-1-3 | DZ515 | Zheng 9023 |
| DZ101 | 98(75)/2-1-2 | DZ519 | Jimai 17 |
| DZ111 | 98(76)/2-2-3 | DZ520 | Jimai 19 |
| DZ117 | 97(98)/1-1-3 | DZ521 | Jimai 20 |
| DZ118 | 97(83)/1-1-1 | DZ522 | Jimai 21 |
| DZ123 | 97(97)/4-2-3 | DZ523 | Jimai 22 |
| DZ130 | 97(24)/5-1-1 | DZ529 | Lunxuan 987 |
| DZ143 | 98(111)/5-2-1 | DZ536 | Luyuan 502 |
| DZ145 | 98(114)/1-1-1 | DZ537 | Yanda 1885 |
| DZ146 | 98(124)2-2-2 | DZ538 | Fengshou |
| DZ152 | 74-25/luofulin 10 | DZ539 | Beinong 2 |
| DZ153 | 75-149/80-25 | DZ541 | Zhongmai 9 |
| DZ154 | 75-144/Hengshui 8116 | DZ547 | Linfen 6010 |
| DZ155 | 75-144/Fengshou | DZ560 | Hengshui 8116 |
| DZ158 | Banong 1403 | DZ565 | Shan 7859 |
| DZ159 | [(170/Jingzuo210) ×80-25] F5 | DZ566 | Jinnong 207 |
| DZ168 | (80A×Gaokang1) F1/(Dao×Jiu)/75-149/Jinan 13 | DZ568 | You8134 |
| DZ169 | (Dao×Jiu)//75-149×9-142/506/80A-2/Jingai 1 | DZ570 | Henong 326 |
| DZ172 | (Xindong 14×Lov10/somon/75-144×Hengshui 8116)F2 | DZ573 | Jinan13 |
| DZ175 | 75-149/(Y83-E4/Honggou×2407) | DZ575 | Jinanhe124 |
| DZ182 | 80-25 | DZ592 | Rusattea |
| DZ185 | 2001（38）/1-2-6 | DZ593 | Asosan |
| DZ186 | 2002（85）/4-1-2 | DZ597 | Atom MY4310 |
| DZ190 | 2001(43)/2-1-4 | DZ598 | Arha MY4270 |
| DZ193 | 2003（28）/4-3-1 | DZ599 | Atgton MY4168 |
| DZ195 | 2003（29）/3-2-4 | DZ600 | Knteh MY6280 |
| DZ196 | 2003（36）/6-3-2 | DZ601 | F181-3 MY5585 |
| DZ199 | 2003（76）/3-11-2 | DZ602 | MY4348 Attnla |
| DZ203 | 2002（41）/4-4-3 | DZ649 | Nongda 212 |
| DZ210 | 2000(115)/2-1-2 | DZ659 | Zhongmai 175 |
| DZ211 | 2000(139)/1-1-1 | DZ680 | Nongda 3488 |
| DZ214 | 2000(8)/1-3-2 | DZ681 | Yumai 34 |
| DZ215 | 2000(16)/2-2-2 | DZ682 | Wanmai 52 |
| DZ217 | 2001(43)/9-2-3 | DZ683 | Xinmai 18 |
| DZ218 | Kendong 03(10) | DZ684 | Yanzhan 4110 |
| DZ221 | Kendong 01(38) | DZ704 | Xiaobaidongmai |
| DZ224 | 2005(1)/7-4-6 | DZ708 | Kuchebaidongmai |
| DZ232 | 2005(62)/15-4-3 | DZ717 | Kayimuhong |
| DZ234 | 2005(65)/7-2-1 | DZ726 | Kadong 4 |
| DZ242 | 2012Hanqu3 | DZ728 | Youpilaina 1 |
| DZ244 | 2012Hanqu5 | DZ729 | Xiaoe 186 |
| DZ247 | 2012Hanqu8 | DZ731 | Aodesa6 |
| DZ250 | 2012Hanqu11 | DZ732 | Xinwukelan 83 |
| DZ255 | 2012Wanqu4 | DZ733 | Xinwukelan 84 |
| DZ256 | 2012Wanqu5 | DZ734 | Aodesa3 |
| DZ257 | 2012Wanqu6 | DZ735 | Wumang 1 |
| DZ259 | 2012Wanqu8 | DZ737 | Afuleer |
| DZ264 | 2012Zaosheng 2(95022-8-1-7) | DZ738 | Zaoyangmai |
| DZ265 | 2006(114)/5-10-2 | DZ740 | Aoweisite |
| DZ266 | 2007Lun2/6-3-1 | DZ742 | Shanqian 2 |
| DZ269 | 2007(6)/13-5-2 | DZ743 | Gaojiasuo |
| DZ270 | 2007(8)/26-2-2 | DZ745 | Huabei 187 |
| DZ271 | 2007(16)/25-5-2 | DZ746 | Huabei 497 |
| DZ272 | 2007(26)/6-1-3 | DZ747 | Beijing 6 |
| DZ274 | 2007(32)/7-4-3 | DZ748 | Beijing 7 |
| DZ275 | 2007(37)/20-1-3 | DZ752 | Jinan 4 |
| DZ277 | 2007(44)/13-2 | DZ754 | Baiyoubao |
| DZ290 | Jihe 124 | DZ758 | Gongnong19 |
| DZ291 | Rusaika | DZ759 | Luofulin 10 |
| DZ292 | Aigton MY4168 | DZ760 | Luofulin 18 |
| DZ299 | Mianyang 31 | DZ761 | Luofulin 13 |
| DZ306 | Kuiyinnan 4 | DZ762 | Cangdong 5 |

All of the accessions originate from different provinces in China.

Table S3 The KASP marker primer sequences

| Marker | Probe | Sequece(5'-3') |
| --- | --- | --- |
| *1A_236720351* | FAM | GAAGGTGACCAAGTTCATGCTTCCTCATCCATTCCTAATGCAA |
| *1A_236720351* | HEX | GAAGGTCGGAGTCAACGGATTTCCTCATCCATTCCTAATGCAG |
| *1A_236720351* | Common | TTGTGGAGGATGTTCGGCAA |
| *1B_473486955* | FAM | GAAGGTGACCAAGTTCATGCTGTGATAGCGGGCATTGACTG |
| *1B_473486955* | HEX | GAAGGTCGGAGTCAACGGATTGTGATAGCGGGCATTGACTA |
| *1B_473486955* | Common | TGTGTATGGAGACGCGGATA |

Table S4 Color traits of two accessions

| Variety | Whiteness | FL* | Fa* | Fb* |
| --- | --- | --- | --- | --- |
| Hongzhitou | 72 | 89.61 | -1.23 | 10.81 |
| Youpilaina | 81.5 | 92.47 | -0.96 | 6.6 |

Table S5 The gene IDs and primer sequences used in real-time quantitative polymerase chain reaction (RT-qPCR)

| Gene ID | Primer F (5'-3') | Primer R (5'-3') |
| --- | --- | --- |
| *TraesCS5D02G013400.1* | TGGACAGCCATGAGAACCTG | GTACTGACCAAAGTTAACGGCG |
| *TraesCS5D02G013100* | CTTCATGTGGAACCCGCTCT | CGTCACGGAGAATCTTGGCT |
| *TraesCS5D02G014300.1* | ACCTTATTCGTGTCCTCGCC | GCAAATGCGATGTCTGTGGG |
| *TraesCS1B02G269100.1* | CGAGTCCGTCATCATCCTCC | GAGCTCTGCCTCGATCAGC |
| *TraesCS1B02G269500.1* | GTTCGACGACGAGCCATCT | CACGGTGTAAAGGGTGGCTA |
| *TraesCS6B02G034100.1* | CTGCTCCAACTCCCACACTT | CCTCGCGGACCTCGTAAG |
| *TraesCS6B02G037000.3* | CAAATTGGGAAGGCAGAGCC | TCCGCAAGCTCTTTGGAATTATC |
| *TraesCS4A02G307200* | ACTTTCTTTGGGTAGTCCGCA | ACAATCTTCAGCAGCACCGA |
| *TraesCS7B02G473400.1* | CGACCCTGCCAGTGCATTAT | CTTCTGCCCGAGGGATGC |
| *Actin* | GGAAAAGTGCAGAGAGACACG | TACAGTGTCTGGATCGGTGGT |

Table S6 Significant SNPs associated with color quality traits by genome-wide association study using BLUP values

| Trait | Marker | Chromosome | Position(bp) | *P*-value | *R^2^*(%) |
| --- | --- | --- | --- | --- | --- |
| FL* | *5B_461134527* | 5B | 461134527 | 1.00E-04 | 5.56 |
|  | *5D_6525346* | 5D | 6525346 | 3.87E-14 | 19.91 |
| Fa* | *1A_236720351* | 1A | 236720351 | 2.01E-05 | 5.52 |
|  | *1A_499212962* | 1A | 499212962 | 8.66E-05 | 5.68 |
|  | *2A_150917099* | 2A | 150917099 | 6.22E-05 | 5.88 |
|  | *4A_46144887* | 4A | 46144887 | 2.91E-05 | 6.36 |
|  | *5A_613539427* | 5A | 613539427 | 7.38E-05 | 5.77 |
|  | *5A_671439068* | 5A | 671439068 | 5.06E-06 | 7.46 |
|  | *5A_671479200* | 5A | 671479200 | 2.67E-05 | 6.41 |
|  | *1B_473235903* | 1B | 473235903 | 6.87E-05 | 5.82 |
|  | *1B_473486955* | 1B | 473486955 | 5.92E-06 | 7.36 |
|  | *1B_474382447* | 1B | 474382447 | 3.79E-05 | 6.19 |
|  | *1B_503482454* | 1B | 503482454 | 6.01E-05 | 5.92 |
|  | *1B_504760661* | 1B | 504760661 | 1.43E-06 | 8.90 |
|  | *3B_665860778* | 3B | 665860778 | 2.14E-05 | 6.59 |
|  | *6B_20151350* | 6B | 20151350 | 3.93E-06 | 7.86 |
|  | *7B_107087295* | 7B | 107087295 | 1.00E-04 | 5.58 |
|  | *4D_238181951* | 4D | 238181951 | 1.80E-05 | 5.58 |
| Fb* | *2A_103633362* | 2A | 103633362 | 9.03E-05 | 5.65 |
|  | *2A_104922253* | 2A | 104922253 | 8.95E-05 | 5.65 |
|  | *2A_117110052* | 2A | 117110052 | 8.07E-05 | 5.72 |
|  | *4A_601242583* | 4A | 601242583 | 9.00E-05 | 5.65 |
|  | *6A_293107295* | 6A | 293107295 | 4.61E-05 | 6.26 |
|  | *4B_148210072* | 4B | 148210072 | 9.67E-05 | 5.61 |
|  | *6B_20151350* | 6B | 20151350 | 6.05E-07 | 9.04 |
|  | *7B_646604247* | 7B | 646604247 | 6.10E-05 | 5.89 |
|  | *5D_6525346* | 5D | 6525346 | 1.90E-05 | 6.62 |
|  | *6D_333490747* | 6D | 333490747 | 9.89E-05 | 5.66 |
| Whiteness | *2A_348674245* | 2A | 348674245 | 2.05E-05 | 7.41 |
|  | *6A_293107295* | 6A | 293107295 | 9.25E-05 | 5.79 |
|  | *5D_6525346* | 5D | 6525346 | 2.24E-11 | 15.57 |

Table S7 *P*-values of *t*-tests for efficacy of different alleles on flour color quality traits

| Trait | Marker | Reference | Allele | | | Number | | | *P*-value | | | |
| --- | --- | --- | --- | --- | --- | --- | --- | --- | --- | --- | --- | --- |
|  |  |  |  |  |  |  |  |  | 2020EM | 2020QT | 2021EM | 2021QT |
| FL* | *5D_6525346* | C | CC | CT | TT | 310 | 7 | 19 | 0.000 | 0.000 | 0.000 | 0.000 |
| Fa* | *1B_473486955* | C | CC | CT | TT | 180 | 18 | 137 | 0.000 | 0.000 | 0.000 | 0.000 |
|  | *1B_503482454* | T | CC | TC | TT | 267 | 9 | 59 | 0.078 | 0.463 | 0.008 | 0.077 |
|  | *5A_671439068* |  |  |  |  |  |  |  |  |  |  |  |
|  | *5A_671479200* | A | AA | AG | GG | 315 | 2 | 18 | 0.001 | 0.001 | 0.000 | 0.002 |
|  | *6B_20151350* | T | CC | TC | TT | 30 | 4 | 290 | 0.009 | 0.007 | 0.001 | 0.003 |
|  | *1A_236720351* | T | TC | TT |  | 58 | 277 |  | 0.000 | 0.000 | 0.000 | 0.000 |
|  | *1B_474382447* | C | CC | CT | TT | 160 | 17 | 158 | 0.000 | 0.000 | 0.000 | 0.000 |
|  | *4D_238181951* | A | AA | AT |  | 281 | 54 |  | 0.000 | 0.000 | 0.000 | 0.000 |
|  | *1B_473235903* | C | AA | CA | CC | 132 | 20 | 182 | 0.000 | 0.000 | 0.000 | 0.000 |
|  | *1B_504760661* | T | AA | TA | TT | 146 | 56 | 122 | 0.000 | 0.001 | 0.000 | 0.000 |
| Fb* | *6B_20151350* | T | CC | TC | TT | 30 | 4 | 290 | 0.001 | 0.007 | 0.007 | 0.008 |
|  | *5D_6525346* | C | CC | CT | TT | 308 | 7 | 20 | 0.000 | 0.000 | 0.000 | 0.000 |
|  | *2A_104922253* |  |  |  |  |  |  |  |  |  |  |  |
|  | *2A_117110052* | C | AA | CA | CC | 286 | 5 | 44 | 0.003 | 0.776 | 0.168 | 0.810 |
|  | *4A_601242583* | C | CC | CT | TT | 229 | 13 | 93 | 0.227 | 0.025 | 0.026 | 0.346 |
|  | *4B_148210072* | C | CC | CT | TT | 314 | 4 | 17 | 0.035 | 0.007 | 0.002 | 0.037 |
| Whiteness | *5D_6525346* | C | CC | CT | TT | 309 | 7 | 22 | 0.000 | 0.000 | 0.000 | 0.000 |
|  | *2A_348674245* | A | AA | AG | GG | 47 | 8 | 264 | 0.010 | 0.018 | 0.012 | 0.026 |
|  | *4A_601242583* | C | CC | CT | TT | 231 | 13 | 94 | 0.025 | 0.061 | 0.000 | 0.302 |

2020EM, 2020QT, 2021EM, 2021QT

Table S8 Genes nearby stable SNPs

| Trait | Marker | Annotation | Candidate gene | |
| --- | --- | --- | --- | --- |
|  |  |  | Former | Latter |
| Whiteness | *5D_6525346* | intergenic | *TraesCS5D02G012300* | *TraesCS5D02G012400* |
|  | *2A_348674245* | intergenic | *TraesCS2A02G244300* | *TraesCS2A02G244400* |
|  | *4A_601242583* | downstream | *TraesCS4A02G307200* | |
| FL* | *5D_6525346* | intergenic | *TraesCS5D02G012300* | *TraesCS5D02G012400* |
| Fa* | *1B_473486955* | intergenic | *TraesCS1B02G269100* | *TraesCS1B02G269200* |
|  | *1B_503482454* | intergenic | *TraesCS1B02G288700* | *TraesCS1B02G288800* |
|  | *5A_671439068* | intergenic | *TraesCS5A02G506500* | *TraesCS5A02G506600* |
|  | *5A_671479200* | intergenic | *TraesCS5A02G506600* | *TraesCS5A02G506900* |
|  | *6B_20151350* | intergenic | *TraesCS6B02G033711* | *TraesCS6B02G033800* |
|  | *1A_236720351* | exonic | *TraesCS1A02G139500* | |
|  | *1B_474382447* | intergenic | *TraesCS1B02G269700* | *TraesCS1B02G269800* |
|  | *4D_238181951* | intergenic | *TraesCS4D02G160600* | *TraesCS4D02G160700* |
|  | *1B_473235903* | intergenic | *TraesCS1B02G268900* | *TraesCS1B02G269100* |
|  | *1B_504760661* | intergenic | *TraesCS1B02G289500* | *TraesCS1B02G289600* |
| Fb* | *6B_20151350* | intergenic | *TraesCS6B02G033711* | *TraesCS6B02G033800* |
|  | *5D_6525346* | intergenic | *TraesCS5D02G012300* | *TraesCS5D02G012400* |
|  | *2A_104922253* | intergenic | *TraesCS2A02G157900* | *TraesCS2A02G158000* |
|  | *2A_117110052* | intergenic | *TraesCS2A02G164500* | *TraesCS2A02G164600* |
|  | *4A_601242583* | downstream | *TraesCS4A02G307200* | |
|  | *4B_148210072* | intergenic | *TraesCS4B02G123900* | *TraesCS4B02G124000* |

Table S9 Annotation of candidate genes

| ID | Start | End | Annotation |
| --- | --- | --- | --- |
| *TraesCS5D02G013400.1* | 7501291 | 7504931 | Lipoxygenase, *LOX2.1* |
| *TraesCS5D02G013100* | 7013571 | 7020162 | LHA1, PMA1 |
| *TraesCS5D02G014300.1* | 8033202 | 8035560 | P450·Gene:*CYP71C7v1* |
| *TraesCS1B02G269100.1* | *473284910* | *473286231* | glutathione transferase |
| *TraesCS1B02G269500.1* | 473864248 | 473868617 | isopentenyl-diphosphate Delta-isomerase, *IPP1* |
| *TraesCS6B02G034100.1* | 20679829 | 20687936 | DExH-box ATP-dependent RNA helicase DExH12，MER3, BRR2A |
| *TraesCS4A02G307200* | 601242852 | 601248654 | —— |

An “——” indicates the missing data.


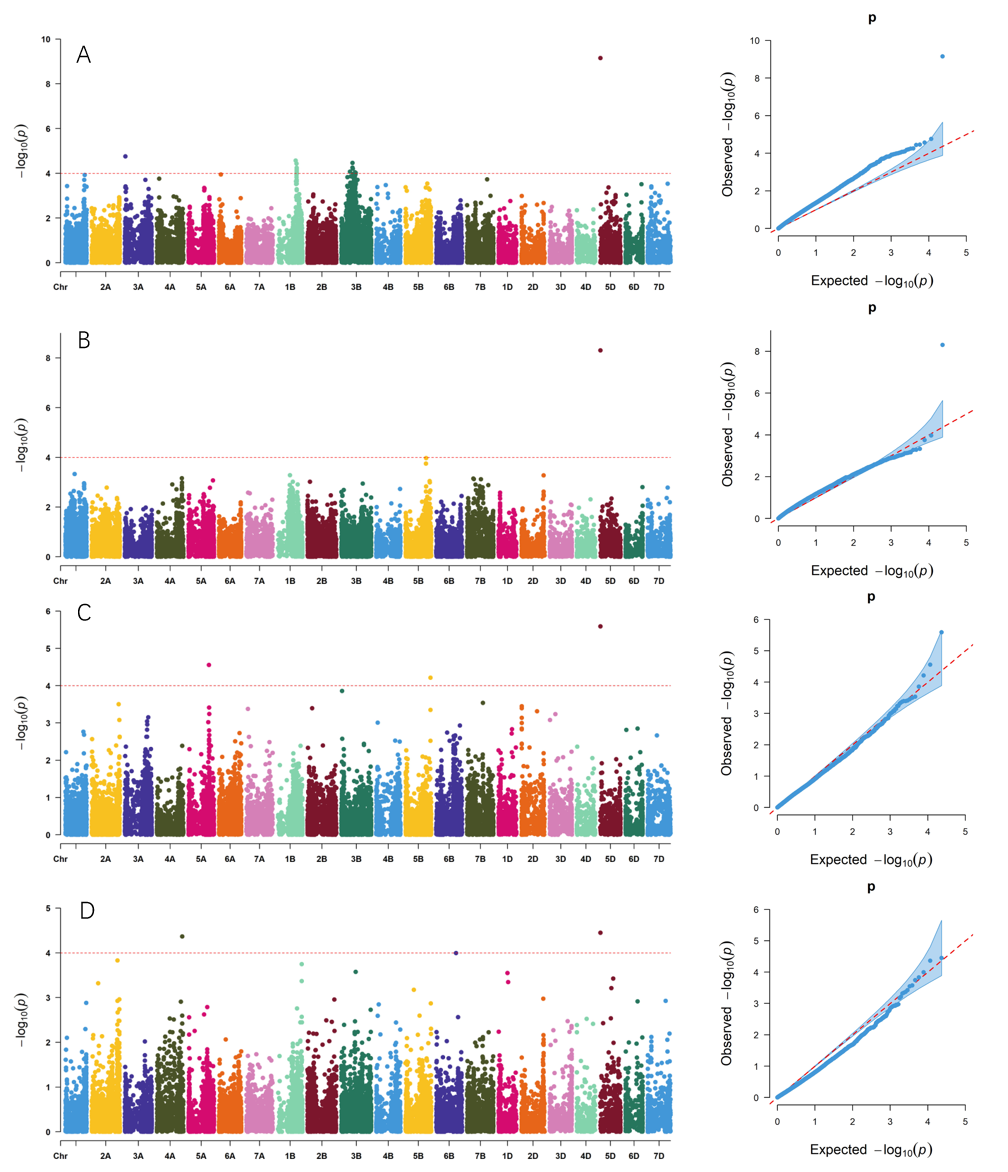


Figure S1 Manhattan and quantile-quantile (Q-Q) plots for FL* identified through genome-wide association analysis in a single environment

A, B, C, D, Manhattan, and Q-Q plots for FL* in 2020EM, 2020QT, 2021EM, and 2021QT, respectively; A horizontal line represents the significance threshold at which markers were considered associated with a trait (*P* < 1E-4, =4).


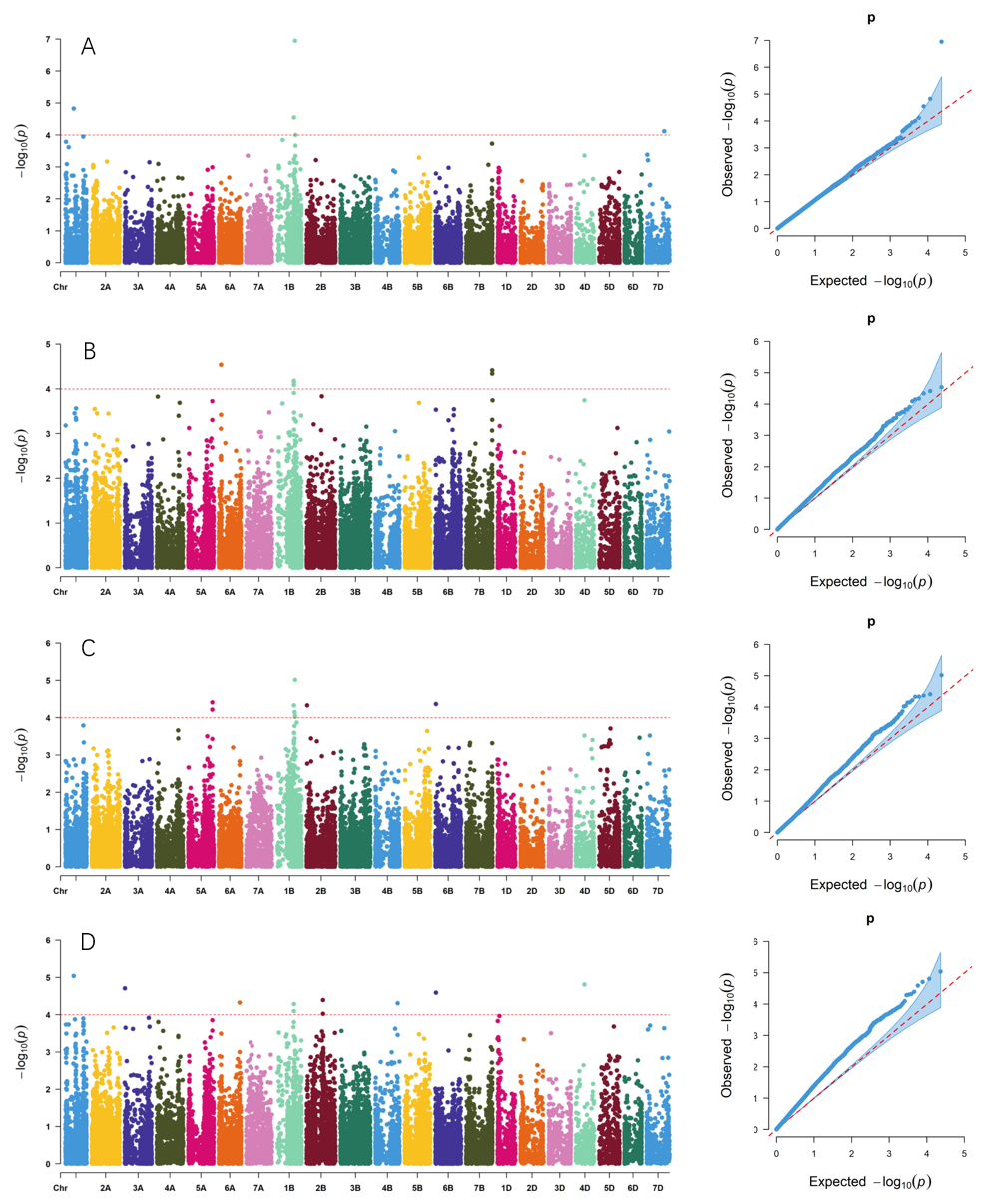
Figure S2 Manhattan and quantile-quantile (Q-Q) plots for Fa* identified through genome-wide association analysis in a single environment

A, B, C, D, Manhattan, and Q-Q plots for Fa* in 2020EM, 2020QT, 2021EM, and 2021QT, respectively; A horizontal line represents the significance threshold at which markers were considered associated with a trait (*P* < 1E-4, =4).


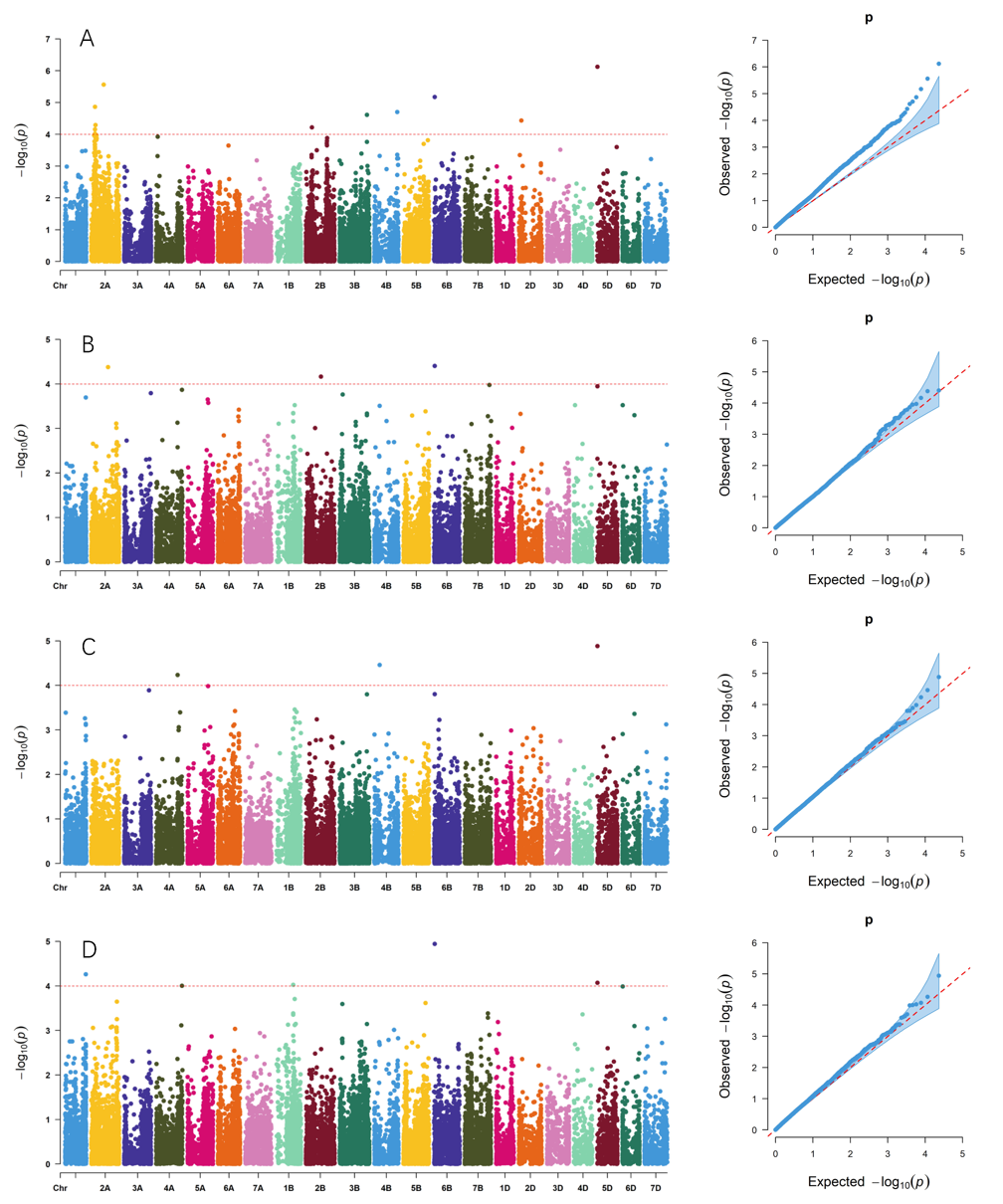
Figure S3 Manhattan and quantile-quantile (Q-Q) plots for Fb* identified through genome-wide association analysis in a single environment

A, B, C, D, Manhattan, and Q-Q plots for Fb* in 2020EM, 2020QT, 2021EM, and 2021QT, respectively; A horizontal line represents the significance threshold at which markers were considered associated with a trait (*P* < 1E-4, =4).


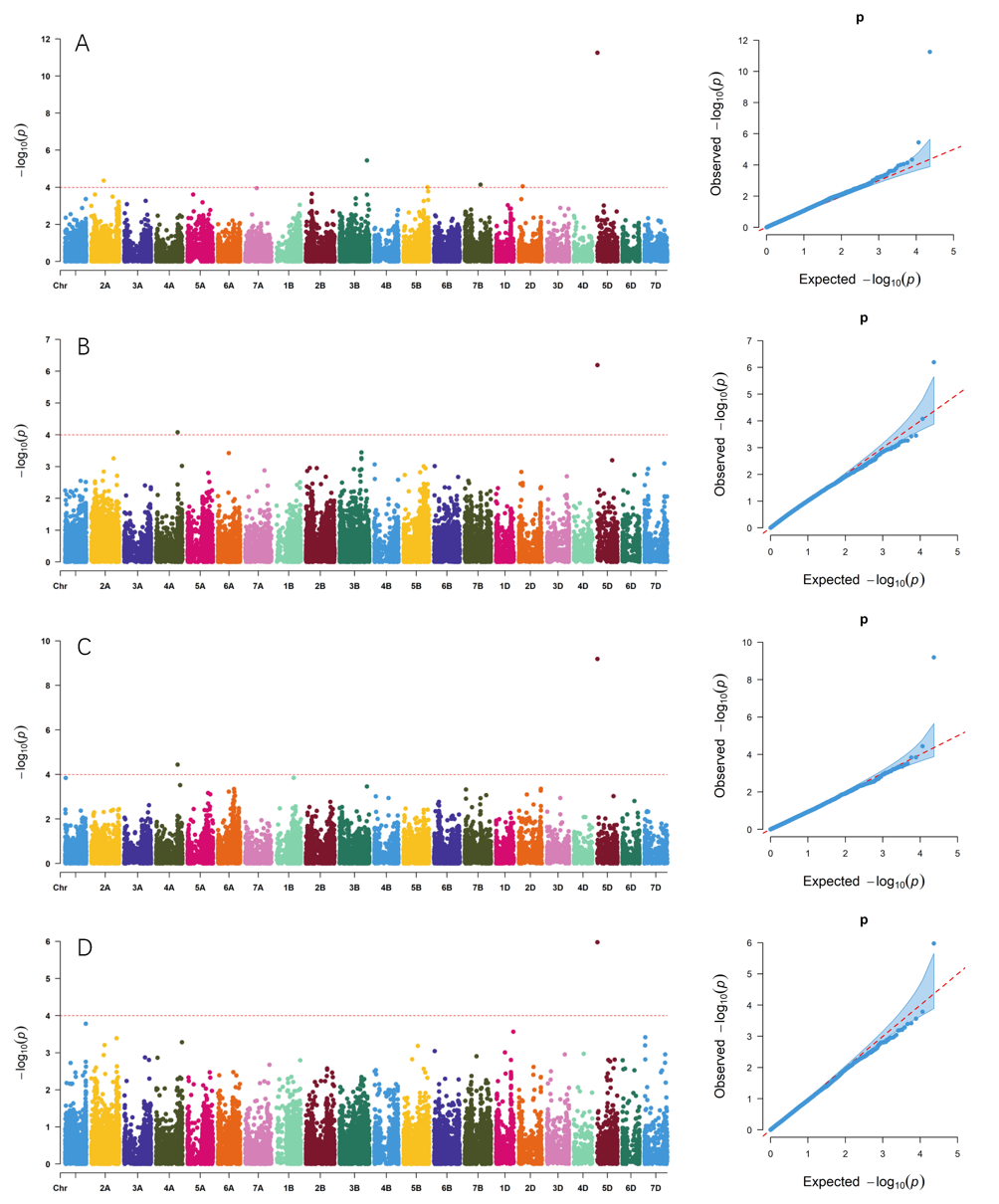
Figure S4 Manhattan and quantile-quantile (Q-Q) plots for whiteness identified through genome-wide association analysis in a single environment

A, B, C, D, Manhattan, and Q-Q plots for whiteness in 2020EM, 2020QT, 2021EM, and 2021QT, respectively; A horizontal line represents the significance threshold at which markers were considered associated with a trait (*P* < 1E-4, =4).


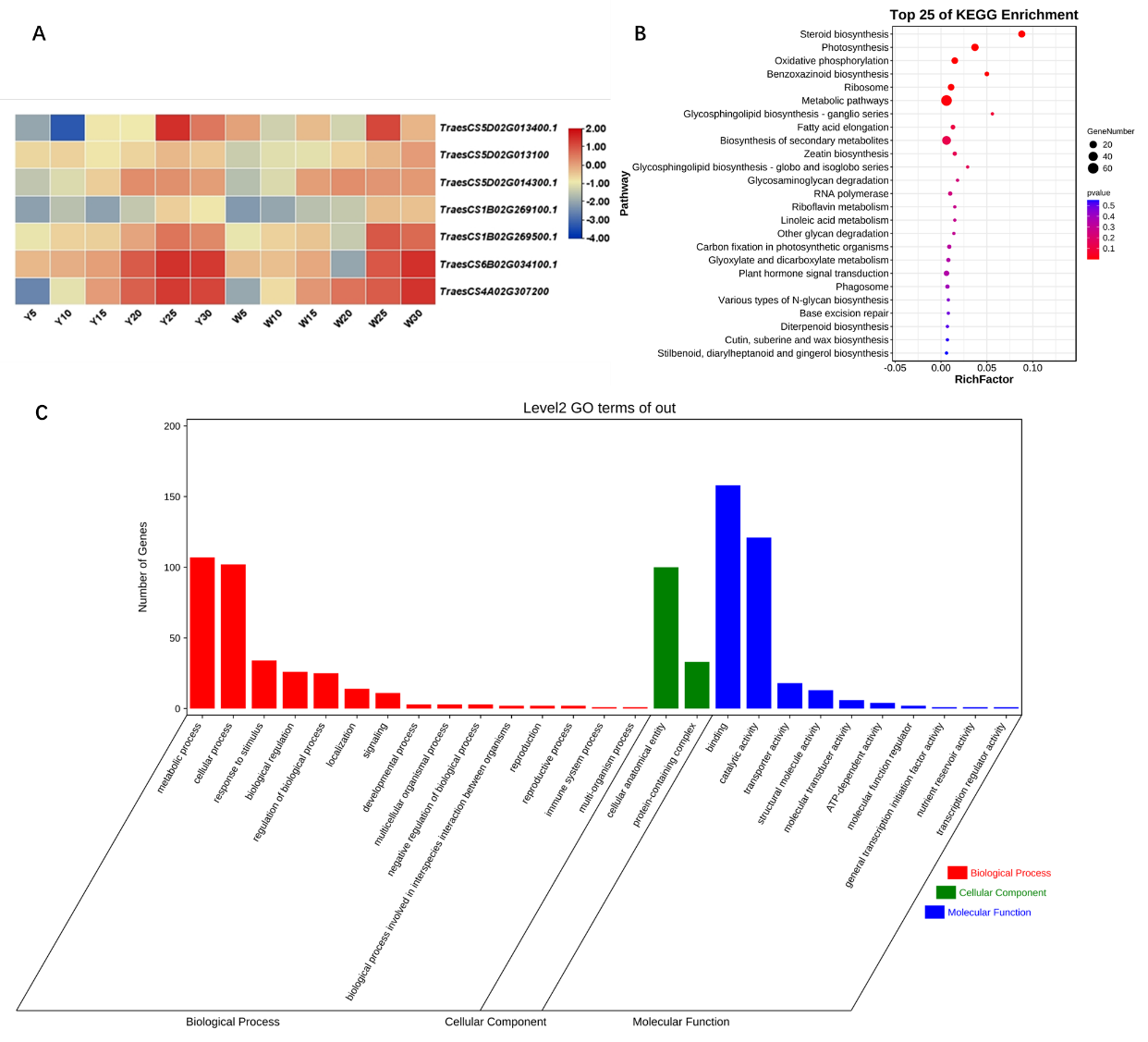


Figure S5 Analysis of candidate genes for flour color quality traits

A, Heat map of candidate genes for color quality traits (Data processed using log 10); "Y" indicates that the flour has a yellowish color and represents the variety as "Hongzhitou"; "W" indicates that the flour has a relatively white color and represents the variety as "Upilaina"; 5, 10, 15, 20, 25, and 30 refer to the days after anthesis. B, KEGG pathway analysis of candidate genes for flour color quality traits. C, GO secondary classification of candidate genes for flour color quality traits.
